# NanoSatellite: accurate characterization of expanded tandem repeat length and sequence through whole genome long-read sequencing on PromethION

**DOI:** 10.1186/s13059-019-1856-3

**Published:** 2019-11-14

**Authors:** Arne De Roeck, Wouter De Coster, Liene Bossaerts, Rita Cacace, Tim De Pooter, Jasper Van Dongen, Svenn D’Hert, Peter De Rijk, Mojca Strazisar, Christine Van Broeckhoven, Kristel Sleegers

**Affiliations:** 1 Neurodegenerative Brain Diseases Group, VIB Center for Molecular Neurology, University of Antwerp-CDE, Universiteitsplein 1, B-2610 Antwerp, Belgium; 2 Biomedical Sciences, University of Antwerp, Antwerp, Belgium; 3 Neuromics Support Facility, Center for Molecular Neurology, VIB - University of Antwerp, Antwerp, Belgium

**Keywords:** Long-read whole genome sequencing, Dynamic time warping (DTW), Variable number tandem repeat (VNTR), ATP-binding cassette, Sub-family A, Member 7 (ABCA7), Alzheimer’s disease

## Abstract

Technological limitations have hindered the large-scale genetic investigation of tandem repeats in disease. We show that long-read sequencing with a single Oxford Nanopore Technologies PromethION flow cell per individual achieves 30× human genome coverage and enables accurate assessment of tandem repeats including the 10,000-bp Alzheimer’s disease-associated *ABCA7* VNTR. The Guppy “flip-flop” base caller and tandem-genotypes tandem repeat caller are efficient for large-scale tandem repeat assessment, but base calling and alignment challenges persist. We present NanoSatellite, which analyzes tandem repeats directly on electric current data and improves calling of GC-rich tandem repeats, expanded alleles, and motif interruptions.

## Background

Half of the human genome is estimated to consist of repetitive DNA elements. These are categorized as interspersed repeats (e.g., *Alu*, *LINE* elements, and segmental duplications) and tandem repeats (TRs). The latter includes short tandem repeats (STRs; a.k.a. microsatellites) which have 1–6-bp motifs and variable number of tandem repeats (VNTRs; a.k.a. minisatellites) with repeat unit length > 6 bp. Compared to non-repetitive DNA, our knowledge on repeats is lagging behind severely, mostly due to technological limitations [1].

Currently, we know of approximately 50 TRs that affect disease, primarily neurological. STRs are particularly involved in rare diseases with high penetrance due to repeat expansions, and VNTRs are mostly associated with common complex disorders [2, 3]. Most often, pathological and benign alleles are categorized based on an arbitrary repeat length cutoff [3]. In reality, however, the disease-associated effects of TRs are more complex. Firstly, one TR can be involved in multiple diseases [2, 3]. Secondly, increasing length of expanded repeats can lead to increased severity of the phenotype or anticipation [3]. Thirdly, repeats can be interrupted by alternative sequence motifs, which influences repeat stability and modifies associated phenotypes [4–8]. Lastly, CpG dinucleotides in TRs can be methylated which may contribute to disease development [9–13].

A comprehensive analysis of TRs therefore requires accurate length estimation, nucleotide sequence determination, and preferably analysis of epigenetic modifications. Unfortunately, the techniques most often used to study TRs in clinical diagnosis and basic research, i.e., Southern blotting and repeat-primed PCR, only provide an estimation of length, with accuracy inversely correlated with repeat size. In addition, these methods only target one TR locus at a time and have high turnaround times. Currently, there is no assay to study TRs simultaneously, let alone on a human genome scale [14].

Next-generation sequencing reads from the most conventional platforms (e.g., Illumina) are too short to directly resolve TRs. In the last 2 years, new algorithms were developed to circumvent this limitation, which enables screening for potential STR expansions. However, these length estimations lack accuracy and validation with gold standard techniques like Southern blotting is still necessary [14].

A solution can theoretically be obtained with long-read sequencing. These technologies have rapidly improved in the last years with both Pacific Biosciences (PacBio) and Oxford Nanopore Technologies (ONT) now providing the opportunity to perform human whole genome long-read sequencing. Long sequencing reads can span entire (expanded) TR alleles, providing TR length, nucleotide composition, and the possibility to detect nucleotide modifications. In practice, however, the higher sequencing error rates of long-read sequencing may limit this application and necessitates evaluation [15]. ONT sequencing has several characteristics which makes it particularly attractive to study TRs. It is based on direct sensing of nucleotides and does not require DNA polymerization. As such, ONT sequencing suffers less from a GC coverage bias in the often GC-rich TRs [16], and nucleotide modifications can be directly detected. Secondly, there is no technical maximum on read length and the sequencing quality does not decay with increasing length [17]. Thirdly, real-time data processing on ONT devices can lower the turnaround time [18]. Lastly, with the release of the high-throughput PromethION sequencing platform in 2018, ONT now provides the most cost-effective human whole genome long-read sequencing option.

A few reports exist on the use of long-read sequencing in TRs. However, these are limited to small-scale tests on plasmids, specifically amplified genomic regions, and/or do not include expanded TR alleles [8, 19, 20]. Only one study attempted whole genome sequencing of an individual with a *C9orf72* repeat expansion; ONT sequencing (on the low-throughput MinION device) yielded 3× coverage without reads spanning the expanded allele [21].

**Fig. 1:**
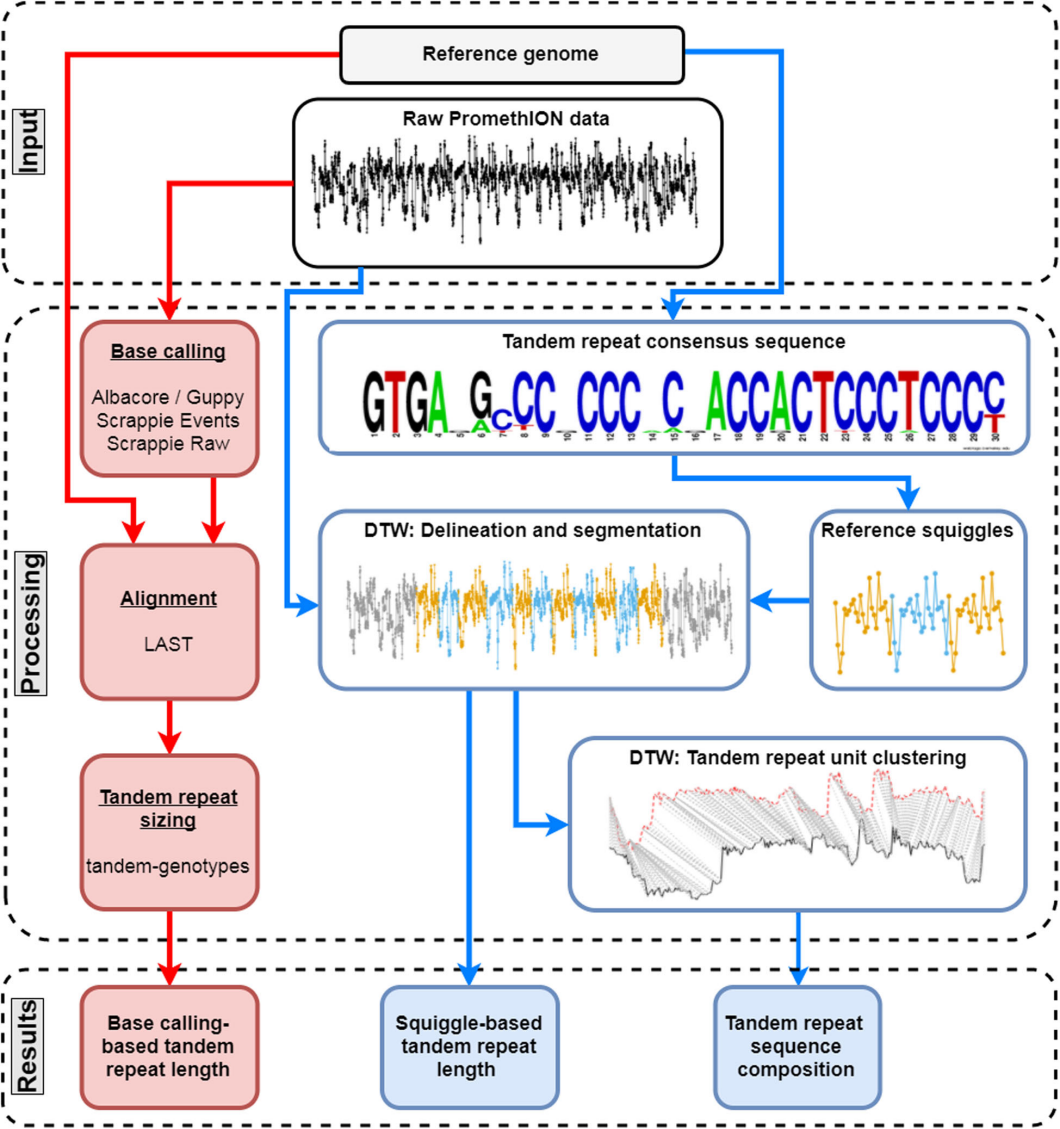
Tandem repeat analysis methods. To extract TR length and sequence information from PromethION data, we used a base calling (red) and our squiggle-based NanoSatellite (blue) approach. Consecutive steps are shown in bold with below the names of the used bioinformatics tools or squiggle illustrations. The “Raw PromethION data” illustration corresponds to a partial PromethION squiggle from a single read spanning the *ABCA7* VNTR. The “TR consensus sequence” figure was obtained from De Roeck et al. [22]. The height of each nucleotide corresponds to its frequency on that position [22]. In the “Reference Squiggles” figure, three *ABCA7* VNTR units (alternating colors) are shown based on Scrappie current estimation. After DTW with raw PromethION data and reference squiggles, the TR is delineated from the flanking sequence and segmented into individual TR units (alternating colors in the “Delineation and segmentation” figure). The final “TR unit clustering” figure depicts the DTW process between two TR units. Each current measurement from a TR unit (red or black) is matched (gray lines) to a current measurement from the other TR unit. More detail of the NanoSatellite process is shown in Additional file 1: Figure S6

The aim of this study was twofold. First, we tested the feasibility and robustness of long-read human genome sequencing on the recently released high-throughput PromethION sequencing device (ONT). Second, we evaluated the use of these data to characterize TR length and nucleotide sequence. We focused our analyses on an *ABCA7* VNTR, for which we recently discovered that expanded alleles are a strong risk factor for Alzheimer’s disease [22]. This VNTR (chr19:1049437-1050028, hg19) has a high GC content, a 25-bp repeat unit with frequent nucleotide substitutions and insertions (consensus motif embedded in Fig. 1), and the total repeat size can reach more than 10,000 bp. Prior to this study, only Southern blotting could be used to approximate the length of this challenging TR.

## Results

### Human long-read genome sequencing with a single flow cell

We sequenced native genomic DNA from 11 individuals on an Oxford Nanopore PromethION sequencing platform. On average, we attained 70,195,516,005 bases (70.2 Gb; ~ 22× genome coverage) output per PromethION flow cell, with a maximum of 98.0 Gb (30.6×) (Table 1). The lowest yields were obtained for Subject09 (43.2 Gb) and Subject10 (48.9 Gb). For these individuals, we respectively used unsheared DNA, or an 8-year-old DNA extraction instead of mechanically fragmented and recently extracted DNA as used in the other sequencing libraries. The final yield of each sequencing run was influenced by the number of available nanopores over time (Additional file 1: Figure S1).

**Table 1:** Summary of individuals included in this study

Individual	*ABCA7* VNTR lengths (kb)		DNA source	PromethION device	Sequencing chemistry	Number of flow cells	Yield (Gb)	Read length N50 (kb)
Subject01	5.1	4.5	QIAmp	α	108	6 + 7^d^	74.4	7.0
Subject02	1.8	1.8	QIAmp	α	108	1	61.3	11.2
Subject03	4.7	2.3	QIAmp	α	109	1	72.5	16.3
Subject04	9.7	0.8	QIAmp	α	109	1	85.5	11.5
Subject05	10.7	1.7	QIAmp	α	109	1	88.5	11.9
Subject06	2.3	0.3	QIAmp	α	109	1	98.0	16.2
Subject07	3.3	3.3	Magtration	α	109	1	77.7	10.6
Subject08	2.3	0.4	Magtration	β	109	1	56.2	14.7
Subject09^a^	5.5	4.3	Magtration	β	109	1	43.2	29.3
Subject10^b^	8.7	2.8	Magtration	β	109	1	48.9	9.1
NA19240	4.5	2.2	Magtration	β	109^c^	5	220.0	15.8

DNA was either extracted with QIAmp (Qiagen) or on a Magtration robotic platform (PSS). An alpha (α) and beta (β) PromethION device were used. Two chemistries were applied: SQK-LSK108 (108) or SQK-LSK109 (109). Yield and mean read length were calculated with NanoPack [23] after Albacore or Guppy base calling. kb kilobases, Gb gigabases, N50 50% of the total sequencing dataset is contained in reads equal or larger than this value. ^a^DNA was not sheared for this individual. ^b^An 8-year-old DNA extraction was used. ^c^SQK-LSK109 was used for four flow cells and for one flow cell was run with SQK-LSK108. ^d^Six debug PromethION flow cells and seven MinION flow cells were used

Overall, the mean read length N50 was 14.0 kb (half of the total number of bases originates from reads with a read length larger than or equal to the N50 value). Sequence length distributions differed between sequencing datasets (Additional file 1: Figure S2). With the exception of Subject10—whose old DNA extraction resulted in shorter reads—read lengths correlated strongly (*R*^2^ = 0.96) with the DNA fragment size prior to ONT library preparation and sequencing (Additional file 1: Figure S3). Overall, the read length was approximately 62% of the average DNA fragment size, which can be attributed to a preferential sequencing of short DNA fragments due to more accessible free DNA ends, unsuccessful repair of ssDNA nicks, and the introduction of dsDNA breaks and ssDNA nicks during library preparation. Apart from Subject01 which we sequenced during the PromethION optimization phase, all sequencing datasets had a similar distribution of quality and an overall median identity to the reference genome of 86% (Additional file 1: Figure S4).

### Base calling-based tandem repeat length and sequence determination

**Fig. 2:**
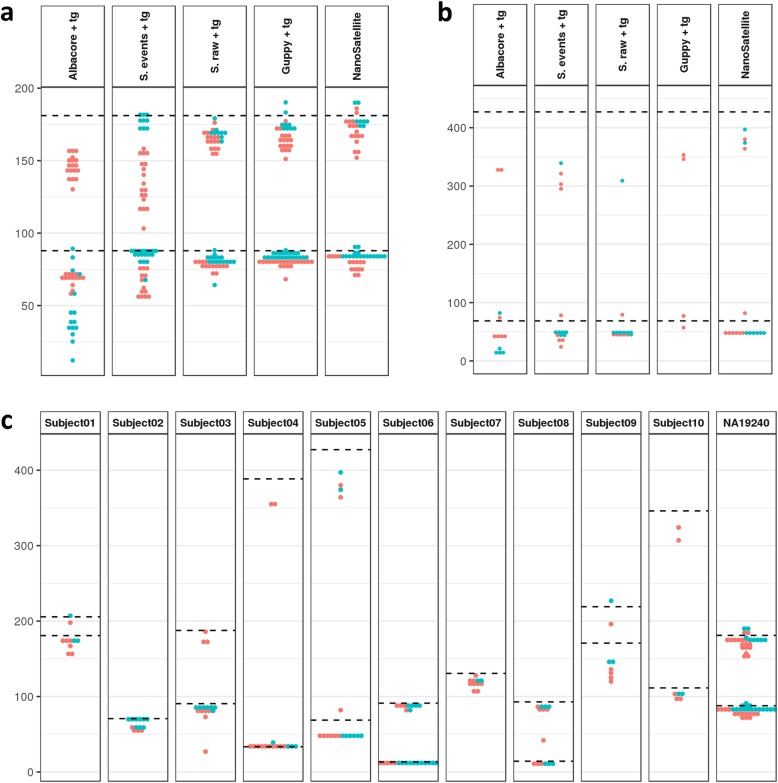
*ABCA7* VNTR length estimates. TR length estimates (the number of TR units is depicted on the *y*-axis) per positive strand (red) or negative strand (blue) PromethION sequencing reads (dots) are shown in comparison to the Southern blotting lengths (dashed lines). **a** Comparison of the five methods (Albacore + tandem-genotypes (tg), Scrappie (S.) events + tg, S. raw + tg, Guppy “flip-flop” + tg, and NanoSatellite) for NA19240, the individual with most sequencing reads. **b** Comparison of the five methods for Subject05, the individual with the largest expanded *ABCA7* VNTR allele. **c** NanoSatellite *ABCA7* VNTR length estimations for all individuals

We evaluated PromethION analysis methods on their ability to resolve different *ABCA7* VNTR lengths varying from 300 bases (~ 12 repeat units) to more than 10,000 bases (~ 400 repeat units), as previously determined by Southern blotting (Table 1). ONT flow cells contain protein nanopores connected to an application-specific integrated circuit (ASIC). As the DNA passes through the pore, nucleotide sequences are shifted, resulting in changes of ionic current that are detected by the ASIC. Each sequenced DNA fragment is therefore represented by a series of current levels, also known as a “squiggle” (illustration embedded in Fig. 1). Conventionally, these squiggles are then base called with the use of neural network-based software [18]. On the one hand, we tested the performance of existing TR analysis methods for which we evaluated three base callers combined with the *tandem-genotypes* algorithm (Fig. 1). We observed that length estimates based on *Albacore—*the original and until recently most commonly used base caller—were underestimated with the largest deviation observed for VNTR spanning reads originating from the guanine-rich negative DNA strand (Fig. 2a), resulting in low accuracy and precision (Table 2). Using the *Scrappie* base caller, with the *Scrappie raw* mode in particular, we observed better TR length estimation accuracy, lower relative standard deviation, and a smaller strand bias effect (Fig. 2a, Additional file 1: Figure S5, Table 2). *Scrappie raw*, however, failed to detect most sequencing reads which span expanded *ABCA7* VNTR alleles (> ~ 229 repeat units) and produced deviating length calls for the few expansion-spanning reads which were detected (Table 2, Fig. 2b, and Additional file 1: Figure S5). Finally, *ABCA7* VNTR length calls based on *Guppy* “*flip-flop*” base calling—which is the recently released successor of *Albacore*—achieved the highest accuracy, lowest standard deviation, and spanned all *ABCA7* VNTR expansions. Read estimations for expanded alleles were better than for other base calling-based approaches (Table 2). These results demonstrate the continuous improvements made on base calling of nanopore sequencing data. Nevertheless, *Guppy* “*flip-flop*” base calling for Subject05 shows a loss of reads originating from the negative strand of the expanded allele, and an overall loss of reads for the shortest allele (Fig. 2b).

**Table 2:** Evaluation of tandem repeat analysis methods on the *ABCA7* VNTR

Method	Accuracy (%)	Relative standard deviation (%)	Number of spanning reads	Expanded read detection (%)	Sequence composition
Albacore + tandem-genotypes	67.3	36.0	154	63	Low consistency
Scrappie events + tandem-genotypes	83.3	14.8	177	88	Low consistency
Scrappie raw + tandem-genotypes	87.7	5.5	181	25	Low consistency
Guppy “flip-flop” + tandem-genotypes	91.2	3.1	176	75	Low consistency
NanoSatellite	90.5	5.6	194	100	High consistency

Accuracy corresponds to the degree of resemblance of the average length estimation and Southern blotting length. The relative standard deviation depicts the spread of length estimates to the mean. The total number of *ABCA7* VNTR spanning reads detected per method is shown under “Number of spanning reads”; shown in more detail in Additional file 1: Table S2. “Expanded read detection” corresponds to the proportion of expanded reads that were detected using the accompanying method compared to the method that detected most expanded reads, with 100% corresponding to the detection of all expanded reads. Sequence composition is based on the resemblance between base called sequences and the *ABCA7* VNTR reference sequence for conventional tools (Additional file 1: Table S1), and the reoccurrence of alternative TR unit patterns in independent sequencing reads for NanoSatellite (Fig. 4)

Subsequently, we evaluated whether we could confidently assess the sequence of the *ABCA7* VNTR. The TR motif was detected in most spanning sequencing reads produced by all four base callers. However, in general, the consensus size was smaller than expected, and substantially more mismatches and indels were observed (Additional file 1: Table S1), which makes reliable TR sequence determination infeasible. *Albacore* in addition produced a VNTR sequence with a strongly diverging sequence composition (Additional file 1: Table S1).

### Novel squiggle-based algorithm to improve tandem repeat length determination

To circumvent errors introduced by base calling and downstream alignment processing steps, we developed “NanoSatellite,” a novel algorithm resolving TRs directly on raw PromethION squiggle data, using dynamic time warping (DTW) (Fig. 1, Additional file 1: Figure S6). DTW is a dynamic programming algorithm to find the optimal alignment between two (unevenly spaced) time series and is also used in other pattern recognition applications, such as speech recognition. TR squiggles were reliably distinguished from the flanking sequence and were subsequently segmented in TR units. Using this approach, we identified more VNTR spanning sequencing reads than the conventional methods described above, and we were able to resolve all VNTR alleles in each sequencing dataset (Fig. 2c). In terms of accuracy, NanoSatellite performed better than *Albacore* and *Scrappie* and approached the accuracy of *Guppy* “*flip-flop*.” The relative standard deviation of NanoSatellite was low, but slightly higher than *Guppy* “*flip-flop*.” In addition, we observed consistent results across all VNTR lengths and DNA strands, with the highest detection rate of expanded VNTR alleles (Fig. 2c, Table 2). For expanded alleles, length calls from both strands were closer to the Southern blotting validated lengths than those predicted by conventional tools (subjects with expanded alleles are shown in Fig. 2b, Additional file 1: Figure S5d and Figure S5i).

### Consistent tandem repeat sequence determination with squiggle clustering

**Fig. 3:**
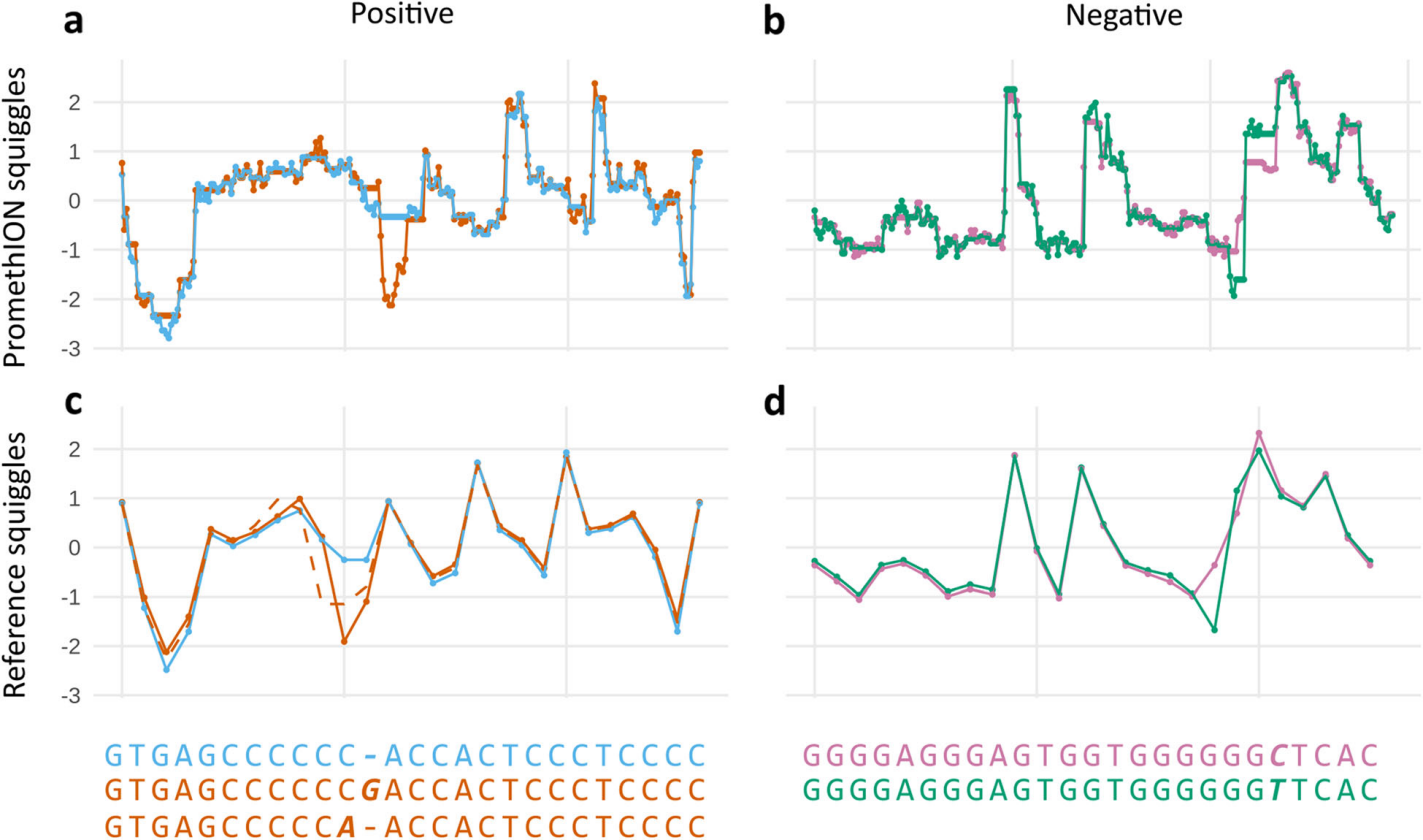
*ABCA7* VNTR squiggle clustering by NanoSatellite. Centroids are shown which were extracted from hierarchical *ABCA7* VNTR squiggle unit clusters originating from positive (**a**) or negative (**b**) DNA strands. Each cluster is shown in a different color. We compared these centroids to positive (**c**) and negative (**d**) reference squiggles with corresponding sequence motifs shown below. The top sequences (blue and purple) correspond to the expected VNTR motifs, while the sequences below (orange and green) contain nucleotide differences (in bold and italic). The alternative (orange) cluster, observed in panel **a**, contains two alternative alleles: a guanine insertion (solid orange line in panel **c**) and a cytosine to adenine substitution (dashed orange line in panel **c**)

We assessed whether alternative sequence motifs can be differentiated on the squiggle level by NanoSatellite. We used unsupervised hierarchical clustering to classify the squiggle TR units. While more alternative sequences may be present, we separated in two clusters per DNA strand, which could be clearly differentiated from each other (Additional file 1: Figure S7 and Additional file 1: Figure S8). Since multiple nucleotides contribute to the current measurement at a given time (i.e., approximately 5 nucleotides for the nanopores used in R9.4 flow cells), a nucleotide change can have different effects on current measurements based on the surrounding nucleotide composition. A base substitution or indel can therefore result in different effect sizes in the positive or negative DNA strand direction. Clustering of positive squiggles was mainly driven by a guanine insertion, or cytosine to adenine substitution at nucleotide ten of the consensus motif (Fig. 3a and Fig. 3c), whereas negative strand clustering was most strongly affected by a cytosine to thymidine substitution at nucleotide 21 (corresponding to the fourth nucleotide in the positive strand) (Fig. 3b and Fig. 3d).

### Alternative sequence motifs can be used for fine-typing VNTRs

**Fig. 4:**
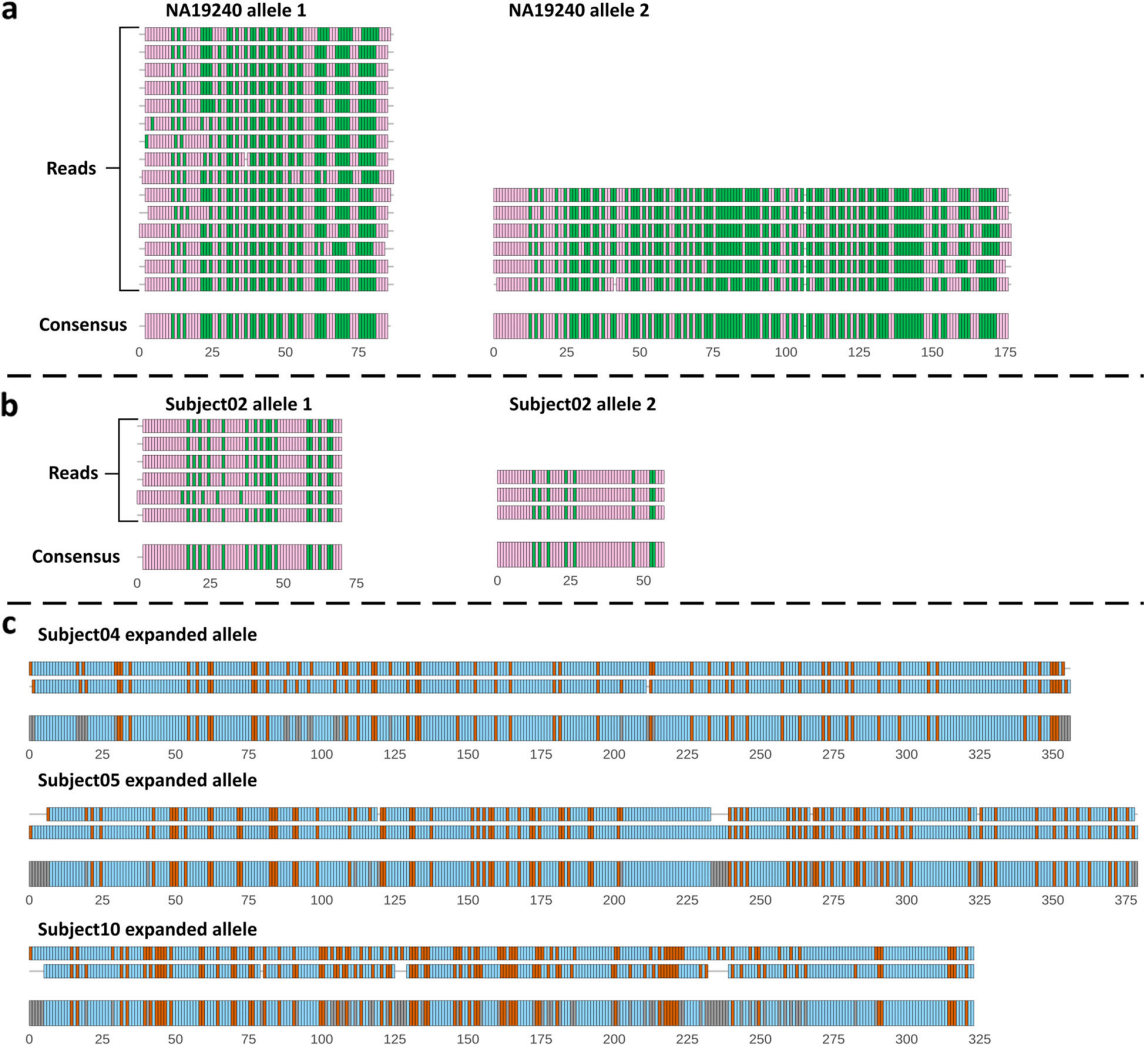
*ABCA7* VNTR sequence reconstruction based on squiggle clusters. Alignments are shown with individual PromethION sequencing reads (narrow segments) and a consensus (broad segments), as annotated in panels **a** and **b**. Each rectangle corresponds to a TR unit. Colors correspond to the TR unit cluster as assigned in Fig. 3. **a** Negative reads originating from the two NA19240 alleles. For both alleles of NA19240, two reads with deviating TR length were removed for clearer visualization. **b** Negative reads corresponding to both alleles of Subject02, for whom a single VNTR length is observed in Southern blotting. **c** Positive reads corresponding to the expanded alleles of Subject04, Subject05, and Subject10

We determined the sequence of the original PromethION reads by ordering the TR unit clusters. We observed a high consistency in clustering patterns between different sequencing reads originating from the same VNTR allele (Fig. 4a). Based on Southern blotting, only one VNTR length was observed for Subject02 and Subject07, compatible with homozygosity. In line with this, squiggle-based length estimation only identified reads corresponding to the Southern blot-based VNTR length (Fig. 2c); however, by examining the read clustering patterns, we could distinguish two alleles that were indeed close in length, but had a different sequence composition (Fig. 4b). While expanded alleles have fewer spanning sequencing reads due to their long lengths, we were able to determine a consistent consensus pattern, which differed between individuals (Fig. 4c).

### NanoSatellite improvements extend to other tandem repeats

**Fig. 5:**
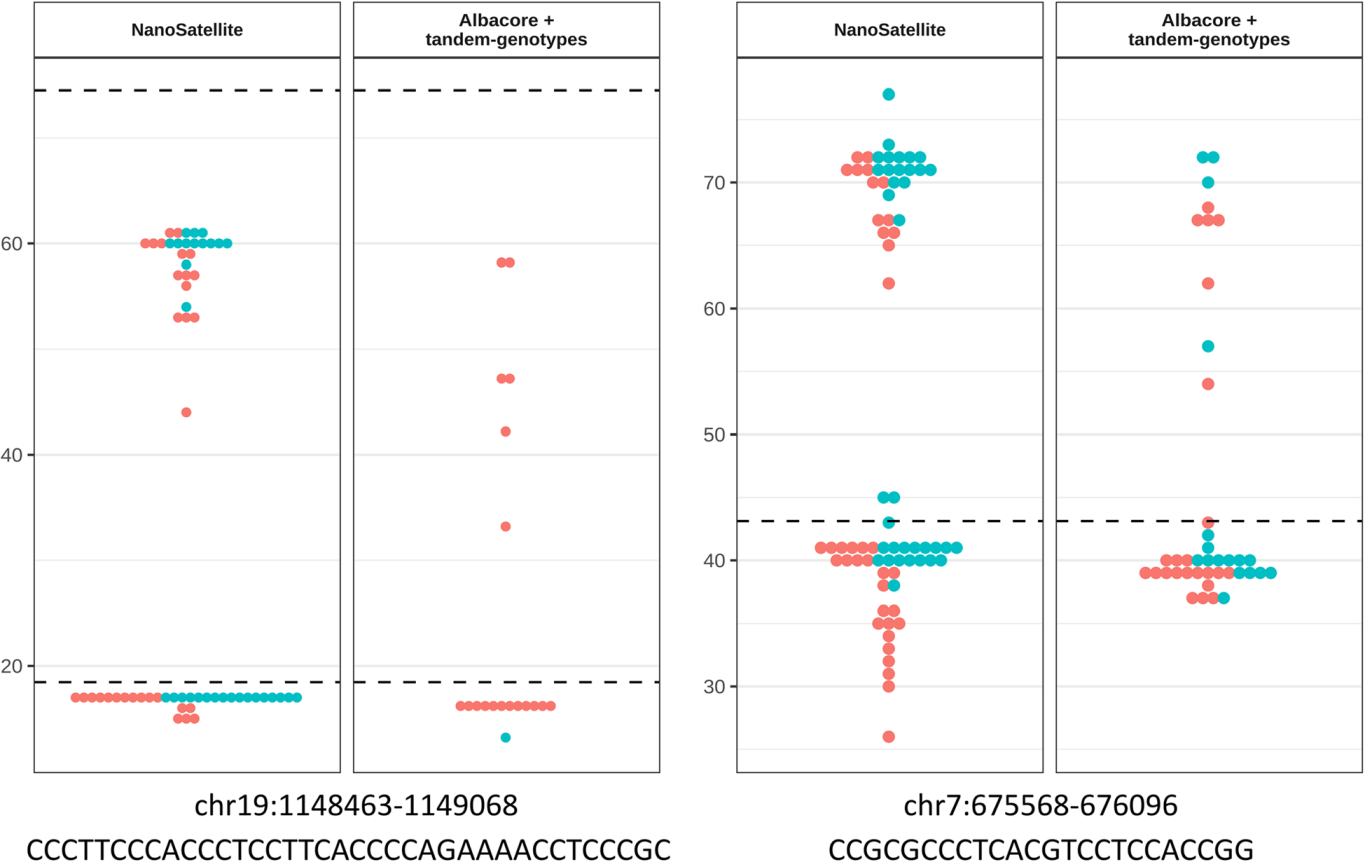
Application of NanoSatellite to other tandem repeats. Comparison of NanoSatellite and Albacore + tandem-genotypes is shown (panels) for two TRs other than the *ABCA7* VNTR, with hg19 genomic coordinates and TR unit motif denoted below. The estimated number of TR units is depicted on the *y*-axis per sequencing read (dots) originating from positive (red) or negative strands (blue). In both examples, NanoSatellite provides a “preferred” outcome as a more concise length call can be made for the largest allele in particular (i.e., read lengths cluster more closely, and more information from both DNA strands is present)

In addition to the *ABCA7* VNTR locus, we assessed whether NanoSatellite enabled improved characterization of other TRs across the genome. We estimated TR lengths with NanoSatellite and tandem-genotypes for 50 highly varying TRs in the PromethION-sequenced NA19240 genome (Table 1). Overall, NanoSatellite uniformly processed sequencing reads originating from both DNA strands, while the tandem-genotype-based method produced strand-biased results with particularly an underrepresentation of guanine-rich sequencing reads (Additional file 1: Figure S9). Based on further comparison of NanoSatellite and tandem-genotypes, we observed four categories: (1) TRs preferentially called with NanoSatellite (*n* = 14), (2) TRs preferentially assessed with tandem-genotypes (*n* = 11), (3) TRs with similar calls by both tools (*n* = 14), and (4) TRs with different results, for which it is not possible to determine which tool provided the “true” length estimates (*n* = 11). For TRs in the first category, NanoSatellite assessment produced more precise clustering of length estimates, better assessment of especially the largest allele, and/or more certainty due to dual DNA strand information. Furthermore, when available, PCR-sized TR lengths resembled those based on long-read sequencing (Fig. 5, Additional file 1: Figure S10). While the overall TR characteristics influencing the performance of TR calling algorithms remain to be explored, we observed a significant difference in GC-content distribution (*p* = 0.004). NanoSatellite particularly performed well for TRs with more than 45% GC content (Additional file 1: Figure S11), most likely due to a loss of guanine-rich sequencing reads after base calling in conventional methods (Additional file 1: Figure S9).

## Discussion

*ABCA7* VNTR expansions result in a > 4-fold increased risk of Alzheimer’s disease [22], yet the technological challenges of investigating TR sequences have so far precluded further research and large-scale screening. Long-read sequencing has the potential to overcome these issues on the condition that sufficient yield and read lengths are consistently obtained to traverse TRs, and algorithms exist that can accurately size even the largest alleles. For the first time, we demonstrate the feasibility of achieving these requirements. We show robust whole genome long-read sequencing yields with up to 98 Gb per flow cell. In addition, we were able to span all *ABCA7* VNTR lengths, including expansions. By combining conventional methods with our newly developed algorithm, we were able to obtain high-quality TR length and sequence determination.

### PromethION sequencing

With an average of 70.2 Gb per PromethION flow cell, we obtained substantially higher yields than the most recently published long-read human genomes. Generation of a 91.2-Gb genome on MinION (ONT) required 39 flow cells (2.3 Gb per flow cell on average) [24], and 225 SMRTcells were required to produce a 236-Gb genome on a PacBio RSII instrument (1.0 Gb per SMRTcell) [25]. The total number of sequencing reads on PromethION varied between sequencing experiments, yet sufficient coverage was achieved for each individual. Yield variance can be attributed to several factors, which we will discuss further along with our recommendations.

First, we observed the lowest yield for the sequencing run with the longest DNA fragments, for which we did not mechanically shear the DNA. For the same amount of DNA, longer fragments result in fewer available free ends to make contact to the nanopores and initiate sequencing. In addition, the available free ends could be masked from the pore by steric hindrance of long DNA molecules. Hence, we opted for shearing of DNA in most DNA libraries. When using sheared DNA libraries, we observed no clear relation between the DNA shearing size (which correlates to the read length) and yield (Table 1); hence, shearing at 20 kb (our highest shearing length) on a Megaruptor (Diagenode) was most optimal in our experience. Second, the amount of DNA loaded on a flow cell makes a difference. Too few molecules result in suboptimal occupation of “open” pores, and too much DNA with attached motor proteins can cause faster depletion of ATP in the sequencing buffer. We adhered to suggested loading amounts as much as possible, yet these recommendations have changed over time. Third, the number of good sequencing pores at the start of sequencing varied due to manufacturing, shipment, and/or storage conditions. Nevertheless, all sequencing runs were performed on flow cells with at least 6000 good pores at the start. Fourth, the rate of pore decline varied, which in turn is determined by several parameters. Interfering chemicals introduced during DNA extraction or library preparation can affect the stability of the flow cell (e.g., detergents can disrupt the membrane in which the nanopores are embedded), pores can collapse, and pores can get blocked. Several efforts by ONT have addressed these issues while this study was in progress including removal of detergents from consumables, brief current reversals during sequencing to unblock pores, and dynamic re-evaluation of “good” sequencing pores instead of fixed 16-h time intervals, as used during the PromethION sequencing runs described in this study. Therefore, we anticipate yield will increase even further in future experiments.

We performed PromethION sequencing using DNA extracted by different methods, sequencing chemistries, and sequencing devices. We observed resembling read quality and similar resolution of *ABCA7* VNTR assessment for all datasets, demonstrating a robust generation of raw sequencing data by the platform. The read length correlated well to DNA fragment size when sequencing fresh DNA extractions. When we used an 8-year-old DNA extraction, however, shorter read lengths were observed. A potential explanation is the introduction of single-strand nicks due to DNA degradation. Library preparation and DNA length determination are not affected by these nicks, since the DNA is kept in a double-stranded conformation. However, during sequencing, single-strand DNA is threaded through the pores and nucleotides after a nick are lost. A DNA repair step during library preparation did not prevent a shorter mean read length for this sample; hence, the use of fresh DNA is recommended. Nevertheless, our method of squiggle-based VNTR length estimation was robust to shorter read lengths.

### Tandem repeat characterization

Expanded alleles of the *ABCA7* VNTR have a strong risk increasing effect on AD, but characterization is restricted by the limitations of Southern blotting [22]. Here, we aimed to provide a better alternative based on PromethION sequencing. We first evaluated the classical sequencing analysis paradigm: base calling of raw sequencing data followed by alignment to a reference genome and ultimately TR variant calling. Base calling of raw ONT sequencing data is based on recurrent neural networks. To obtain very high accuracy, these machine learning algorithms require comprehensive training datasets which are often imperfect and can result in reduced base calling accuracy [15]. Several approaches exist to improve accuracy such as Nanopolish [26]; however, all of these require the aid of a reference genome, which is not available for (expanded) TRs. The lack of representative reference genomes also hinders reliable alignment of reads with TR sequences. Concerning TR length, we observed low accuracy and precision for *Albacore* and *Scrappie events* base callers. *Scrappie raw* obtained better results in this context, but failed to traverse most expanded VNTR alleles, which are crucial from a clinical point of view. *Guppy* “*flip-flop*” base calling performed the best in terms of accuracy and precision and performed better for expanded alleles, but still lost important (expanded) TR spanning reads for specific individuals (Fig. 2b), potentially caused by the downstream alignment and TR calling steps. On a nucleotide level, the erroneous base calls precluded reliable detection of alternative TR unit motifs. Particularly, *Albacore* produced a deviating sequence composition, which has been observed in other GC-rich TRs [21].

To overcome these issues, we developed NanoSatellite, a DTW-based algorithm to perform TR variant calling directly on the raw squiggle level. We observed a strong correlation between Southern blotting lengths and repeat lengths estimated from the PromethION sequencing reads. The small differences in length estimation between both technologies can in part be explained by the limited resolution of Southern blotting. In addition, squiggle-based estimates had high precision and performed well across all sequencing lengths, particularly expanded VNTRs. Moreover, NanoSatellite allowed investigation of TR nucleotide composition through direct comparison of raw TR unit squiggles instead of derivative base calls. We were able to observe clear differences in squiggle signals that could be attributed to nucleotide substitutions and insertions. Sequencing reads originating from the same VNTR allele showed highly consistent patterns of (alternative) TR unit squiggle motifs, hence validating that this squiggle-based method can be used to determine the sequence of TRs. Based on these sequences, we observed two VNTR alleles for individuals that appeared to have only one fragment on Southern blotting, thereby confirming that we spanned all VNTR alleles in all sequenced individuals.

When comparing NanoSatellite and tandem-genotypes, we noted that overall, NanoSatellite was able to produce more length estimates and was less strand biased. A high calling rate of spanning sequencing reads, as obtained with NanoSatellite, enables robust quantification of TRs with low-to-medium coverage, which is currently the norm for whole genome long-read sequencing. We subsequently tested the applicability of both tools on other highly variable TRs across the genome. Sequence characteristics (e.g., composition, repeat unit size, number of repetitions) can be highly variable between TRs, affecting the DNA-nanopore kinetics, raw data, and downstream analysis. Our findings underscore that the analysis strategy to call TR lengths may need to be tailored to the characteristics of the TR of interest. NanoSatellite generally performed better on TRs with higher GC content and is particularly useful for the detection of long TR alleles, which are often most clinically relevant. A limitation of this comparison is the lack of a “truth” dataset for the verification of the TR genotype calls. In contrast to the *ABCA7* VNTR, experimentally validated TR lengths are difficult to come by, since Southern blotting is very labor intensive, especially when assessing larger numbers of TRs simultaneously, and PCR amplification is often hindered by the AT-rich or GC-rich nucleotide compositions and large sizes of these TRs. Additionally, sequencing of synthetic plasmids with known TR lengths could serve as a partial validation, but does not convey the complexity of whole genome sequencing (e.g., they do not contain alternative TR units, are generally shorter than truly expanded alleles, nucleotides are not modified, and no repetitive elements are present in the flanking sequences). Nevertheless, we can make use of the degree to which TR length estimations from different sequencing reads cluster together in two alleles, particularly when length estimates from both DNA strands overlap. Dense clusters of lengths correspond well with validated lengths, while diffuse length estimates and/or different clusters per strand indicate inaccuracies, as observed by us and other researchers [20]. The tested TRs contained relatively large motifs, which are generally more stable and reduce the odds for somatic mutations which would invalidate this clustering approach. Furthermore, for several TRs, we see that both tools produce similar length calls, and since they are based on very different data processing algorithms, this provides more certainty that the TR lengths estimated by long-read sequencing approach the truth. TRs for which we obtained PCR validation showed a strong concordance between PCR-sized lengths and estimates from long-read sequencing data, further validating this approach.

NanoSatellite requires only genome coordinates of a TR of interest to generate reference squiggles. The TR delineation, segmentation, clustering, and sequence reconstruction are executed in an unsupervised fashion with a minimum of arbitrary cutoffs. To achieve the highest sequence determination accuracy, we limit the analysis to biclustering on the strongest squiggle differences. Further sub-clustering to identify more TR motif interruptions and potentially nucleotide modifications is possible, yet requires supervised decisions and validation to balance the number of detected variants and accuracy. For TRs with very small motifs, interruptions could interfere with pattern recognition. In general, the tolerability to nucleotide changes in the TR is inversely correlated to the motif size. Nucleotide substitutions in the *ABCA7* VNTR for instance are well tolerated, and NanoSatellite can be used to confidently call these alternative TR units. The same substitutions, however, will have larger effects on squiggle patterns of very short motifs. Whether such a nucleotide change interferes with the pattern recognition, with less accurate TR length estimation as a consequence, further depends on the TR sequence and type of change (i.e., nucleotide transitions generally produce less pronounced differences than transversions).

Since NanoSatellite is based on a completely new paradigm, the algorithm is not as time-optimized as conventional variant calling tools. We therefore recommend to first use conventional tools (with Guppy “flip-flop” base calling in particular) for genome-wide TR analysis and follow-up with NanoSatellite for those TRs which are not showing accurate results, specifically in case of a high GC content, a good motif length to nucleotide change ratio, and suspicion of large expansions. To facilitate further development, the algorithm is freely available and written in the statistical programming language R, which provides strong support of DTW functionality. We achieved high single-read accuracy for both TR length and sequence, which opens novel avenues in TR research. Many TRs in the human genome—some of which are currently uncharacterized—can be studied at once with a single sequencing run and somatic differences of unstable (expanded) TRs could be evaluated, which eventually will lead to the identification of novel disease-associated TRs and improved diagnostics.

## Conclusions

In this study, we establish the use of long-read sequencing of multiple human genomes with a single sequencing run per individual, achieving up to 98-Gb output per PromethION flow cell. Furthermore, we developed an algorithm to study TRs on a raw squiggle level which provides a second opinion to existing algorithms and can overcome some of the problems which are inherent to base calling and alignment, such as inefficient calling of expanded TR alleles and inconsistent determination of TR sequence composition. For all datasets—even those with a relatively low yield, or relatively low mean read length due to DNA fragmentation—we were able to accurately determine repeat length for both *ABCA7* VNTR alleles, which ranged from 300 bp to more than 10,000 bp. The robust performance and high yield of single PromethION sequencing run combined with the high accuracy and precision of our novel algorithm suggest that future high-throughput detection and screening of TRs such as the *ABCA7* VNTR is attainable, both for research and clinical purposes.

## Materials and methods

### Study population

We performed long-read whole genome sequencing on DNA from 11 individuals (Table 1), using the PromethION sequencing platform (Oxford Nanopore Technologies (ONT), Oxford, UK). Ten individuals (Subject01 till Subject10) were recruited in the context of the Belgian Neurology (BELNEU) Consortium [22, 27] and consisted of AD patients (*n* = 6), an FTLD patient, a family member at risk of developing dementia, and healthy elderly control individuals (*n* = 2). All participants and/or their legal guardian provided written informed consent for participation in genetic studies. The study protocols were approved by the ethics committees of the Antwerp University Hospital and the participating neurological centers at the different hospitals of the BELNEU consortium and by the University of Antwerp. For all individuals, Epstein-Barr virus transformed lymphoblastoid cell lines (LCLs) were available. In addition, we included the previously described NA19240 PromethION sequencing dataset [28]. *ABCA7* VNTR lengths were determined in all individuals with Southern blotting as previously described [22].

### DNA preparation for PromethION sequencing

LCL were cultured with 1640 RPMI medium (Thermo Fisher Scientific, Waltham, USA), supplemented with 15% fetal bovine serum, 2 mM l-glutamine, 1 mM sodium pyruvate, 100 IU/mL penicillin, and streptavidin. Cell counting was performed on a Luna II (Logos Biosystems, South Korea) automated cell counter. Five million cells per individual were then resuspended in PBS. To establish the optimal protocol for DNA preparation for PromethION sequencing, different procedures for DNA extraction and fragmentation were tested. Extraction of DNA was done according to the manufacturer’s protocol with either QIAmp DNA Blood mini spin columns (Qiagen, Hilden, Germany) or automated extraction on a Magtration 8LX platform (Precision System Science (PSS), Matsudo, Japan) (Table 1). RNA degradation was carried out with RNase A during cell lysis in the QIAmp extraction protocol, and after completion of the Magtration protocol.

The extracted DNA was fragmented to a mean length of 15 kb or 20 kb with Megaruptor (Diagenode, Liège, Belgium), with the exception of Subject09 and three aliquots of NA19240 which remained unsheared. All DNA was size selected on BluePippin (Sage Science, Beverly, USA), with a high pass protocol retaining all fragments above a minimum size cutoff, which ranged from 7 to 10 kb. DNA was subsequently purified with Agencourt AMPure XP beads (Beckman Coulter, Brea, USA) in a 1:1 volume ratio. DNA size analysis was conducted on a Fragment Analyzer with DNF-464 High Sensitivity Large fragment 50 kb kit, as specified by the manufacturer (Agilent Technologies, Santa Clara, USA).

### ONT library preparation and sequencing

Different sequencing set-ups were applied as outlined in Table 1 due to frequent improvements of ONT sequencing chemistry, flow cells, and PromethION sequencing devices.

We followed the “1D Genomic DNA by Ligation sequencing on PromethION” protocol (Oxford Nanopore Technologies) according to the latest sequencing kit version (SQK-LSK108 or SQK-LSK109), with slightly increased incubation times during end preparation, purification, and final elution to increase yield. Briefly, DNA was first repaired and dA-tailed with NEBNext FFPE DNA Repair Mix (New England Biolabs (NEB), Ipswich, USA) and NEBNext End repair/dA-tailing (NEB). This was either performed serially (SQK-LSK108) or in a single step (SQK-LSK109). Purification was done with AMPure XP beads. Subsequently, ONT adapters were ligated to the DNA library with the NEB Blunt/TA Ligase Master Mix (SQK-LSK108) or with the NEBNext Quick Ligation Module and the ONT supplied “Ligation buffer” (SQK-LSK109). AMPure XP beads were then used for clean-up together with ONT’s “Adapter Bead Binding Buffer” (SQK-LSK108) or ONT’s “L (ong) Fragment Buffer” (SQK-LSK109). DNA was eluted in the supplied Elution Buffer and loaded on all four inlets of a primed PromethION flow cell.

The flow cells (FLO-PRO001) were either part of an initial debug phase for early PromethION optimization or commercially available flow cells. For Subject01, six debug flow cells were used, while the other samples were sequenced on a single commercial flow cell, with the exception of NA19240 for which the goal was to obtain very high genome coverage depth by using multiple flow cells (Table 1). Upon arrival, all flow cells were subjected to quality control and were used for sequencing within a week. With the exception of debug flow cells, all flow cells used for sequencing had at least 6000 available pores.

During the course of this study, sequencing was conducted on two PromethION devices: an alpha and a beta unit. The main difference between both was the incorporated GPU computational module in the beta device, which allowed real-time base calling.

During the PromethION optimization phase, we also sequenced DNA from Subject01 on seven MinION flow cells (Table 1) to compare performance of the DNA extractions and library preparations on both platforms. The “1D Genomic DNA by Ligation sequencing on MinION” protocol was followed using SQK-LSK108 chemistry, R9.4 flow cells (FLO-MIN106), and a Mk1 MinION platform (MIN-101B).

### Data analysis

Albacore (ONT) was the first base caller available to the public and, until recently, the most widely used. Recently, ONT released Guppy, which is the successor to Albacore. Early versions of Guppy (< v2.3.5) were similar to Albacore. Later versions (≥ v2.3.5) include a new and more accurate base calling model, which is called “flip-flop” base calling. Guppy is furthermore optimized for faster computation on GPU and embedded in beta PromethION sequencing devices. Lastly, ONT also provides the developmental base caller Scrappie (https://github.com/nanoporetech/scrappie), which has two modes of base calling: “events” and “raw.” All PromethION sequencing reads were first processed using conventional base calling and genome alignment procedures: data from the alpha and beta PromethION devices were respectively base called with Albacore v2.2.5 (ONT) and Guppy v1.4.0 (ONT); since both correspond to very similar base calling results, they were grouped under the term “Albacore” in the results of this manuscript. Subsequent alignment was performed with minimap2 [29] with hg19 (GRCh37) as the reference genome. NanoPack was used to summarize experiment metrics [23].

#### ABCA7 VNTR analysis using existing methods for tandem repeat sizing

For the purpose of comparison of existing methods for TR length determination, reads aligning to a 100-kb genome region containing the *ABCA7* VNTR and flanking sequences (chr19:1000000-1100000) were re-base called with Albacore (v2.2.5), Scrappie events (v1.3.1-f31cada), Scrappie raw (v1.3.1-f31cada), and the recently released Guppy (v2.3.5), which employs a “flip-flop” base calling model (Fig. 1). The latter base caller is referred to as “Guppy flip-flop” in this manuscript. Alignment was then carried out on each of the four base called datasets using LAST v941 [30] with base caller-specific trained LAST parameters. Calculation of repeat length was performed with tandem-genotypes [20]. Tandem-genotypes estimates the number of TR units as the difference in length between sequencing reads and the reference sequence, divided by the consensus repeat unit size. To determine absolute repeat units per sequencing read, we added the number of TR units in the reference (23.2 for the *ABCA7* VNTR) defined by Tandem Repeats Finder (TRF) [31]. Computation was parallelized using gnu parallel [32].

We evaluated the ability of sequencing reads processed by these three base callers to resolve TR sequence composition. All reads spanning the *ABCA7* VNTR (according to tandem-genotypes analysis) were processed with the TRF algorithm [31] using lenient parameters to account for base calling errors: a matching weight of 2, mismatching penalty of 3, indel penalty of 5, match probability of 80, indel probability of 10, and minimum alignment score of 14. To determine the *ABCA7* VNTR pattern per sequencing read, we considered repeats with a pattern size between 18 and 32 bp and selected the repeat with the highest copy number. Next, per base caller and DNA strand, we counted the number of reads with an *ABCA7* VNTR pattern and calculated average sequence composition metrics.

### Squiggle-based *ABCA7* VNTR data analysis

Existing methods for TR length determination as described above depend on base calling and alignment accuracy; both of which are often suboptimal in low-complexity and/or repetitive sequences. To overcome these challenges, we designed “NanoSatellite,” a novel pattern recognition algorithm, which bypasses base calling and alignment and performs direct TR analysis on raw PromethION squiggles (Fig. 1, Additional file 1: Figure S6). Most of this method is based on consecutive rounds of dynamic time warping (DTW). DTW is used in many different applications, such as speech recognition and analysis of electrocardiograms, and it is also the main constituent of “Read Until,” an algorithm designed to enrich sequences of interest on an Oxford Nanopore sequencing platform by quickly identifying patterns at the squiggle level [33].

#### Code and data availability

All code is freely available on GitHub (https://github.com/arnederoeck/NanoSatellite). All *ABCA7* VNTR spanning raw current PromethION fast5 files are publicly available via study PRJEB29458 on the European Nucleotide Archive (https://www.ebi.ac.uk/ena/data/view/PRJEB29458). Data accession to NA19240 sequencing data is reported in De Coster et al. [28].

#### Generation of reference squiggles

To enable pattern recognition on raw PromethION data, we first translated DNA nucleotide sequences to an estimated squiggle pattern. We used TRF to delineate TRs of interest in the genome (chr19:1049437-1050028 for the *ABCA7* VNTR). Next, we extracted 250 bp of flanking sequence on both sides of the TR. We converted the consensus TR motif defined by TRF (GTGAGCCCCCCACCACTCCCTCCCC for the *ABCA7* VNTR), as well as the flanking sequences and their respective reverse complements to approximate squiggles using the “squiggle” module of Scrappie (v1.3.1-f31cada).

#### Tandem repeat delineation

Using the reference squiggles, TR length determination was performed on raw PromethION data with DTW by first finemapping the TR boundaries and subsequently segmenting the TR into individual TR units. First, we selected TR spanning reads for which tandem-genotypes was able to estimate TR lengths and we included reads aligning on both sides of the TR after minimap2 alignment. Raw squiggle data was extracted from the original fast5 files using the rhdf5 package [34] in R [35]. The extracted current levels were split in overlapping windows. These windows and the reference squiggles were then z-scale normalized. DTW of strand-matched flanking sequence reference squiggles and windowed sequencing reads was performed using the dtw R package [36] with both sides unfixed. Since each nucleotide is represented by one data point in reference squiggles and multiple current measurements in raw PromethION data, we applied the Minimum Variance Matching (MVM) algorithm, with an MVM step pattern of 25 (each reference squiggle data point is allowed to match 25 current measurements or less) [37]. A distance measure is generated for each DTW alignment, with lower distances corresponding to higher similarity between two time series. We selected the windows with the lowest DTW distance (i.e., the raw PromethION squiggle data which matches best with the reference squiggle) and fine mapped the TR start and end with DTW using a reference squiggle composed of flanking sequence and multiple TR units.

#### Tandem repeat segmentation

Subsequently, the delineated PromethION TR squiggle was split up in individual TR units using a strand-matched reference squiggle composed of five TR units. DTW with a 25 MVM step pattern was started at both ends of the TR with one end fixed and an open end towards the center of the TR. In both sets, three TR units were defined, after which the process was repeated with the fixed end matching the last defined unit of the previous cycle, until both sets crossed each other. Overlapping segmentations were resolved by choosing the segmentation with the lowest DTW distance. The average distance from TR segmentation in all sequencing reads was recorded. Sequencing reads with an average distance larger than 1.5 times the interquartile range from the 75th percentile were discarded.

#### Tandem repeat unit clustering and sequence determination

For the purpose of identification of the underlying nucleotide sequence, all TR unit squiggles were clustered using the dtwclust package [38] in R [35]. Strand-specific distance matrices were calculated with symmetric DTW distances (the “cost” of matching two raw squiggles, each corresponding to a TR unit, where a high distance indicates low similarity in sequence composition between two TR units, and vice versa). Subsequently, TR units were clustered using hierarchical clustering with Ward’s method. A visual representation of the TR unit variability is shown by heatmaps and dendrograms in Additional file 1: Figures S7 and S8. For each strand, we created two clusters, based on the first two branches of the dendrogram. Centroids for each cluster were extracted using the partition around medoids (PAM) method. To determine the corresponding TR unit sequence, we compared the centroids to reference squiggles of known alternative VNTR motifs based on TRF analysis of the *ABCA7* VNTR in the reference genome. We reconstructed the sequencing reads as a chain of TR unit clusters and created a consensus sequence through alignment with the msa package [39].

### Tandem repeat statistics and visualization

We evaluated TR length estimation of the three tested base callers combined with tandem-genotypes and the squiggle approach by calculating several metrics for each. All TR length estimations by PromethION sequencing reads (*r*_*a*, *i*_,) were assigned per method and per subject to a TR allele *a*, with *i* ranging from 1 to the total number of reads per allele (*n*_*a*_). The average length of all reads per allele is denoted as $\overline{r_a}$ with standard deviation *s*_*a*_, and the Southern blotting length per allele as *L*_*a*_. The accuracy (the degree to which PromethION length estimations correspond to Southern blotting length estimation) per allele corresponds to $\left(1-\frac{\left| La-{\overline{r}}_a\right|}{L_a}\right)\times 100\%$ and a method-specific accuracy was calculated by taking the median of all allele accuracies. As a measure for precision (corresponding to the closeness of estimates from individual reads originating from the same allele) we determined the relative standard deviation per allele as $\frac{s_a}{\ {\overline{r}}_a}\times 100\%$ and calculated the method-specific median. Alleles with less than 2 reads in one of the four analysis methods were not included in these accuracy and precision calculations. All visualizations were made with ggplot2 [40] in R [35].

### NanoSatellite characterization of other tandem repetitive loci

We evaluated the characterization of other TRs based on ONT sequencing, using NanoSatellite on the one hand, and a “conventional” Albacore base calling—LAST alignment—tandem-genotypes assessment as described above on the other hand (Fig. 1). We selected TRs based on the Tandem Repeats Finder algorithm [31], embedded in the UCSC genome browser “Simple Repeats” track, which met the following criteria: match percentage more than 90%, period size between 20 and 40 nucleotides, and more than 15 copy numbers in the hg19 reference genome. The resulting 1113 TRs were first characterized in the publicly available NA19240 genome (Table 1) with tandem-genotypes, which outputs the results according to decreasing differences compared to the reference genome [20]. The top 50 TRs were assessed with NanoSatellite (Additional file 1: Table S3). To support the detection of diploid TR alleles, we used *k*-means clustering with two centers in R and calculated the median, 25th percentile, and 75th percentile for each cluster. For both NanoSatellite and tandem-genotypes, we assessed the ability to resolve both DNA strands, separation of diploid alleles, and clustering of length calls. TRs were subsequently classified based on whether NanoSatellite and tandem-genotypes produced similar or divergent calls. For the latter, we indicated the “preferred” genotyping method by manually scoring the TR length estimation plots (e.g., Figure 5 and Additional file 1: Figure S10). We evaluated how well read lengths from both strands clustered together and supported the presence of diploid alleles, aided by unsupervised *k*-means clustering as described above. When both tools provided divergent calls, TRs were classified as “Inconclusive” if a plausible genotype could not be determined (Additional file 1: Table S3). The differences in distribution of GC content, match percentage, period size, and total length of the TR were subsequently assessed between these categories (Additional file 1: Figure S11). For TRs for which we scored a better performance of NanoSatellite (the “NanoSatellite preferred” category), we attempted PCR amplification followed by gel electrophoresis to validate the TR lengths. PCR optimizations for all TRs were performed with the Expand Long Template PCR System (Sigma-Aldrich, St. Louis, USA) and LongAmp Taq (NEB), with and without the addition of betain, at annealing temperatures ranging from 45 to 65 °C and with sufficiently long elongation times to support efficient amplification of long DNA sequences. To confirm specificity, the PCR amplicons were Sanger sequenced using BigDye reagents (Thermo Fisher Scientific) on a 3730 DNA analyzer (Thermo Fisher Scientific). Confidently sized PCR lengths were added to Fig. 5 and Additional file 1: Figure S10.

## Supplementary information

**Additional file 1.** Supplementary figures and tables

**Additional file 2.** Review history.

## Data Availability

The datasets supporting the conclusions of this article are available in the European Nucleotide Archive repository, PRJEB29458, https://www.ebi.ac.uk/ena/data/view/PRJEB29458 [41]. All NanoSatellite code is freely available on GitHub (https://github.com/arnederoeck/NanoSatellite) [42] under the open source license: MIT. The NanoSatellite release which was used in this article is deposited on zenodo with DOI: 10.5281/zenodo.3357940 (10.5281/zenodo.3357940) [43].
