# Supplementary material for: NanoSatellite: accurate characterization of expanded tandem repeat length and sequence through whole genome long-read sequencing on PromethION
Source: Genome Biol. 2019 Nov 14;20:239. doi: 10.1186/s13059-019-1856-3 (PMC6857246; doi:10.1186/s13059-019-1856-3)
Supplement: Supplementary file 1 — Additional file 1. Supplementary figures and tables [file 13059_2019_1856_MOESM1_ESM.docx]

Additional data: NanoSatellite: accurate characterization of expanded tandem repeat length and sequence through whole genome long-read sequencing on PromethION.

Arne De Roeck^1,2^, Wouter De Coster^1,2^, Liene Bossaerts^1,2^, Rita Cacace^1,2^, Tim De Pooter^3^, Jasper Van Dongen^1,2^, Svenn D’Hert^3^, Peter De Rijk^3^, Mojca Strazisar^3^, Christine Van Broeckhoven^1,2^, and Kristel Sleegers^1,2^

1 Neurodegenerative Brain Diseases group, Center for Molecular Neurology, VIB - University of Antwerp, Antwerp, Belgium
2 Biomedical Sciences, University of Antwerp, Antwerp, Belgium
3 Neuromics Support Facility, Center for Molecular Neurology, VIB - University of Antwerp, Antwerp, Belgium

^*^Corresponding author: Prof. Dr. Kristel Sleegers MD PhD

Neurodegenerative Brain Diseases Group; VIB Center for Molecular Neurology

University of Antwerp - CDE

Universiteitsplein 1, B-2610, Antwerp, Belgium

Email: kristel.sleegers@uantwerpen.vib.be

| **a** | 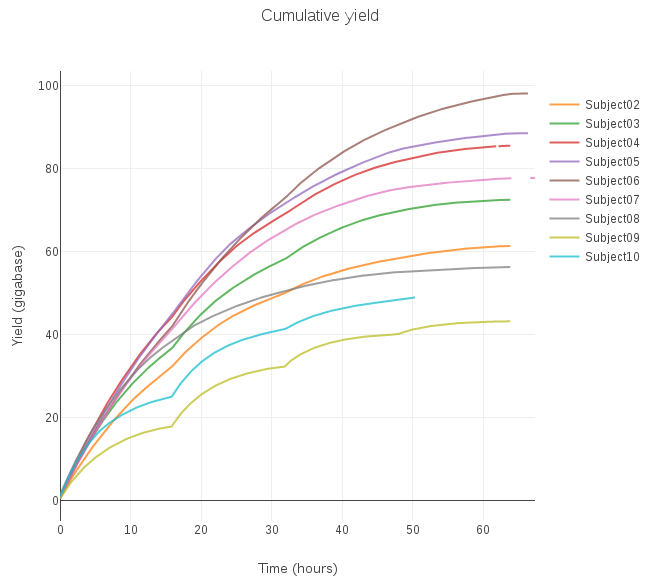 |
| --- | --- |
| **b** | 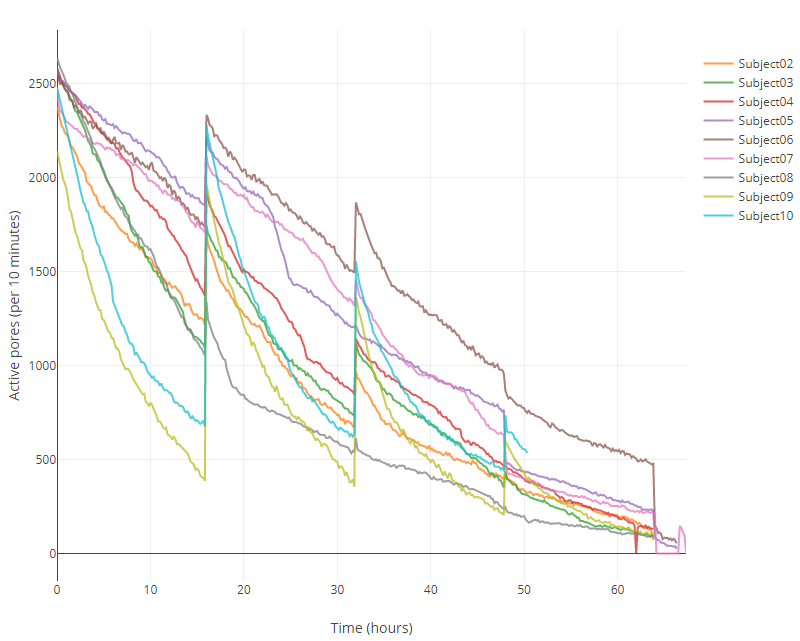 |

**Figure S1: Cumulative yield and pore usage during PromethION sequencing. (a)** The cumulative number of sequenced nucleotides (y-axis) is shown over time (x-axis) for all individuals sequenced on a single flow cell. **(b)** The corresponding number of pores producing nucleotide sequences within a 10 minute interval (y-axis) is shown. During a sequencing run, the number of nanopores capable of processing DNA diminishes, resulting in slower accumulation of sequencing reads. Every 16 hours, the available pores on the flow cells were re-evaluated during the so called “mux scans”, which can lead to sudden improvements of sequencing nanopores and yield. Overall, sequencing runs lasted approximately 64 hours. We manually aborted sequencing of Subject10 after 50 hours, since the flow cell was no longer producing meaningful amounts of data. These graphs were produced with NanoPack. Subject01 and NA19240 are not included, since data for each originated from multiple flow cells.


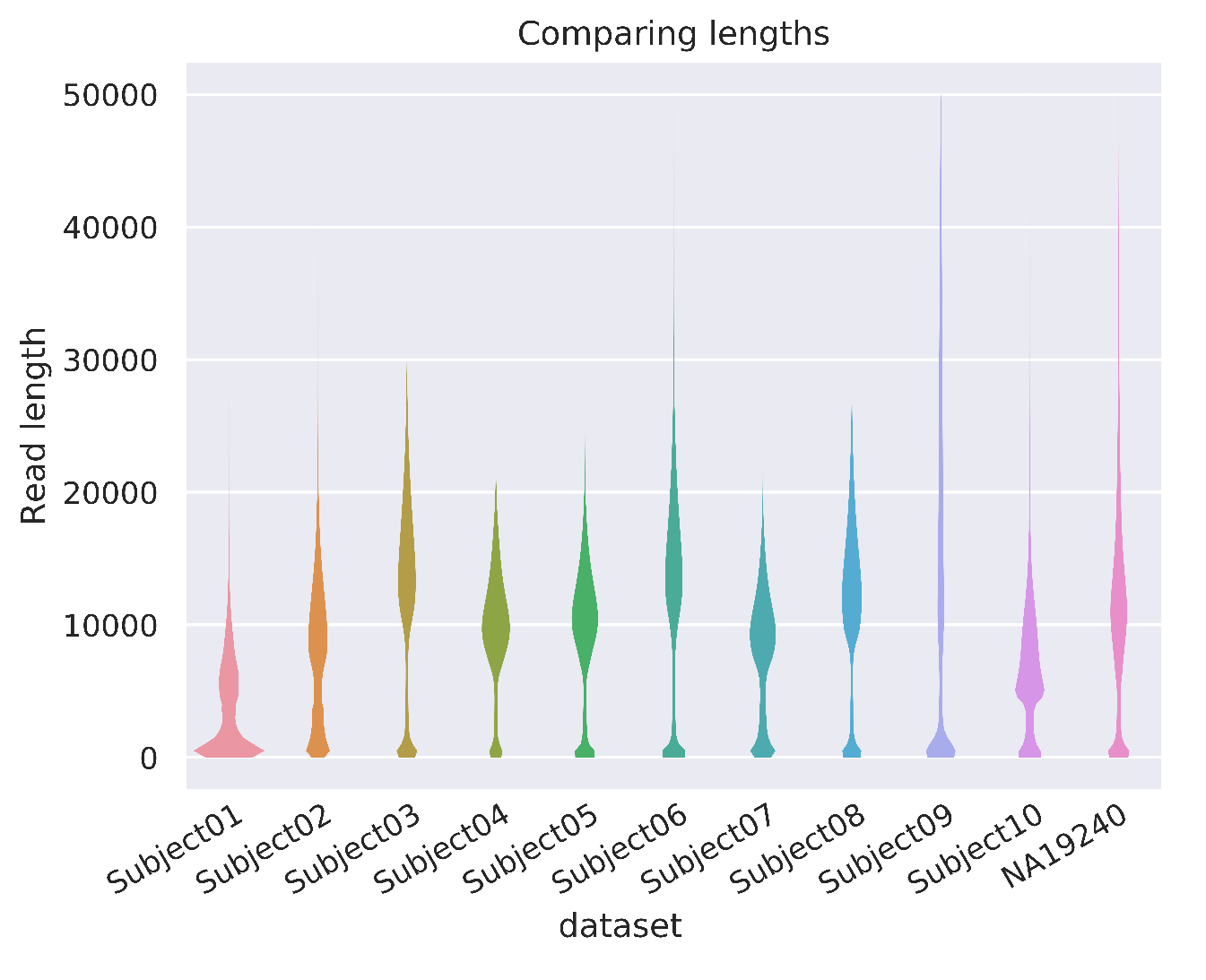


**Figure S2:** Violin plot distributions of read lengths for each PromethION sequencing dataset with a cut-off at 50kb, produced with NanoPack.


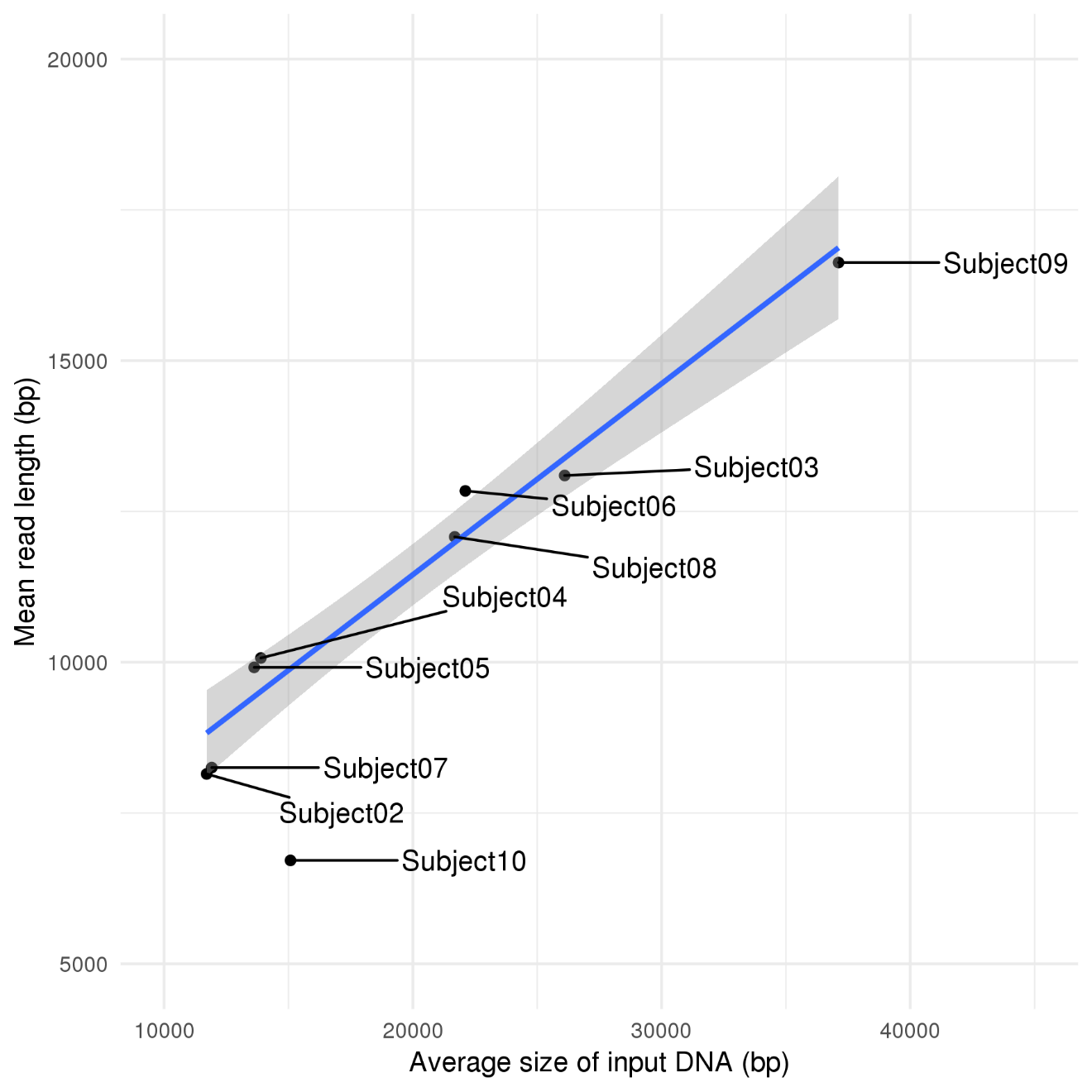


**Figure S3: Comparison of DNA fragment length and read length.** After DNA extraction, fragmentation and size selection, and before ONT library preparation and sequencing, we measured the average DNA fragment length (x-axis) on a Fragment Analyzer (Agilent Technologies), using accompanying DNA smear analysis. We compared these values to the mean read length, as calculated by NanoPack. A trend line (blue) with standard error (gray area) are also shown. With the exception of Subject 10 (for whom an old DNA extraction was used), DNA fragment length and mean read length correlated strongly (R^2^ = 0.96). Absolute values of both metrics differ from each other since a DNA smear analysis favors long DNA fragments. Subject01 and NA19240 are not included, since data for each originated from multiple library preparations and flow cells.


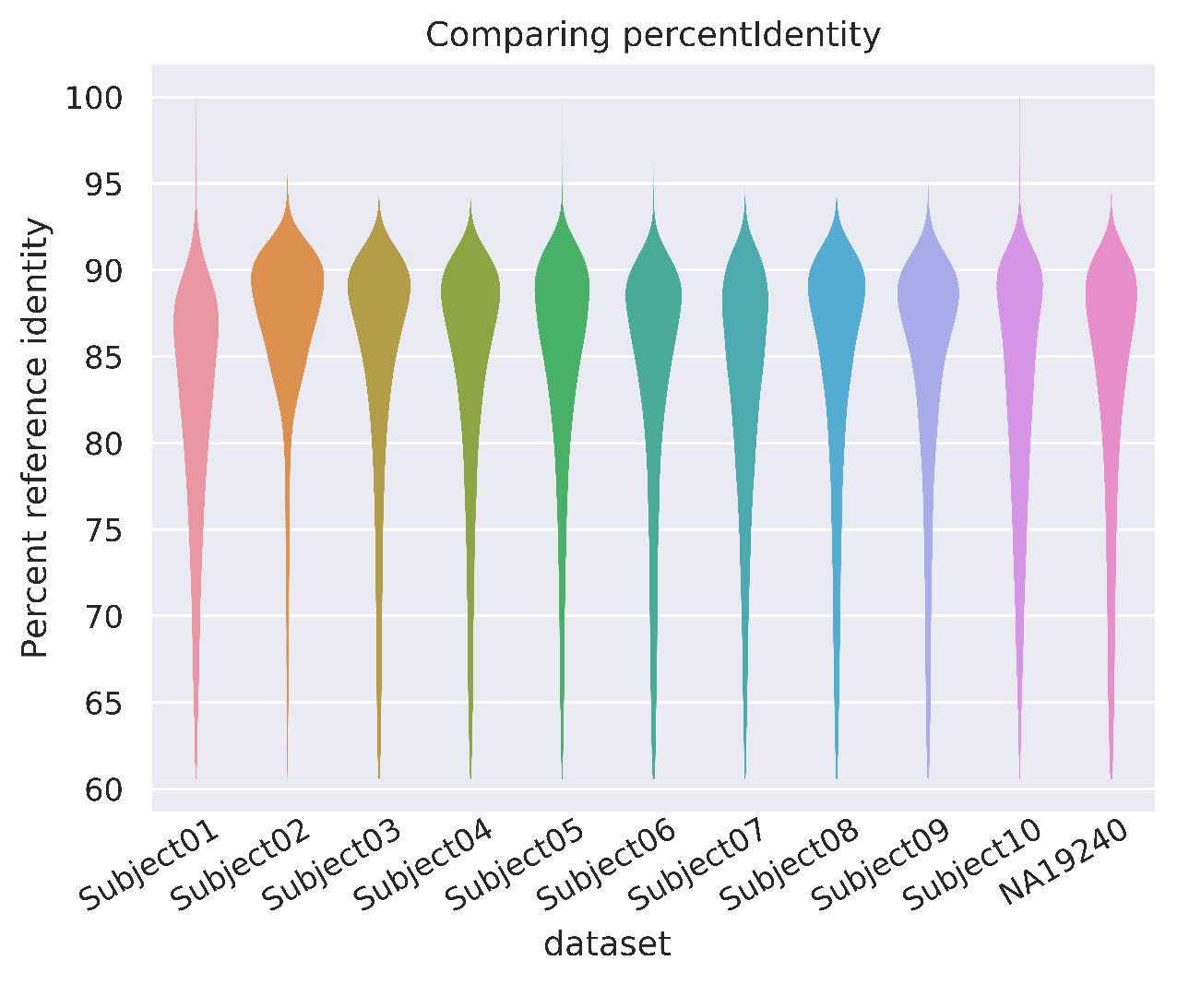


**Figure S4:** Violin plot distributions of the percent reference identity (the sequence similarity between aligned reads and the corresponding reference genome) for each PromethION sequencing dataset, generated with NanoPack.

| **a) Subject01**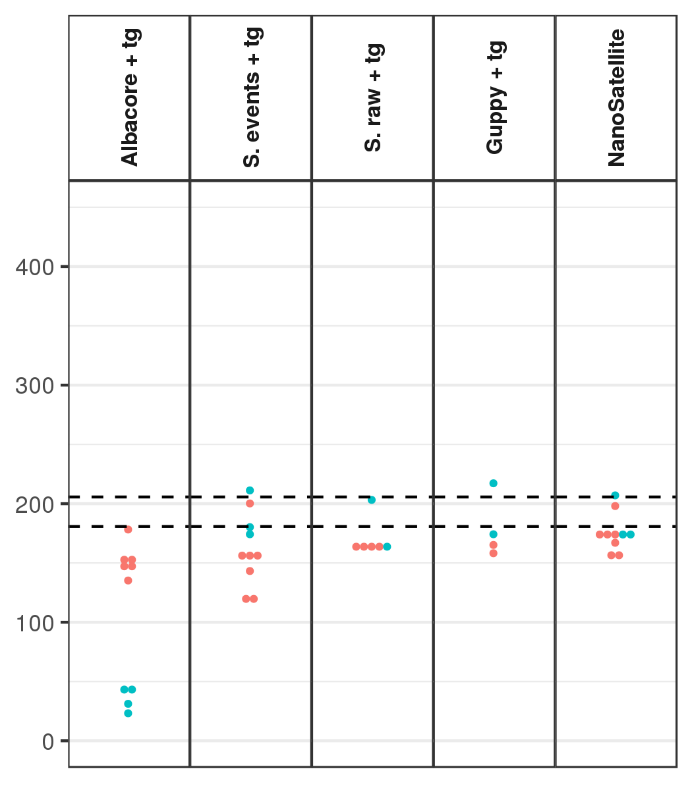 | **b) Subject02**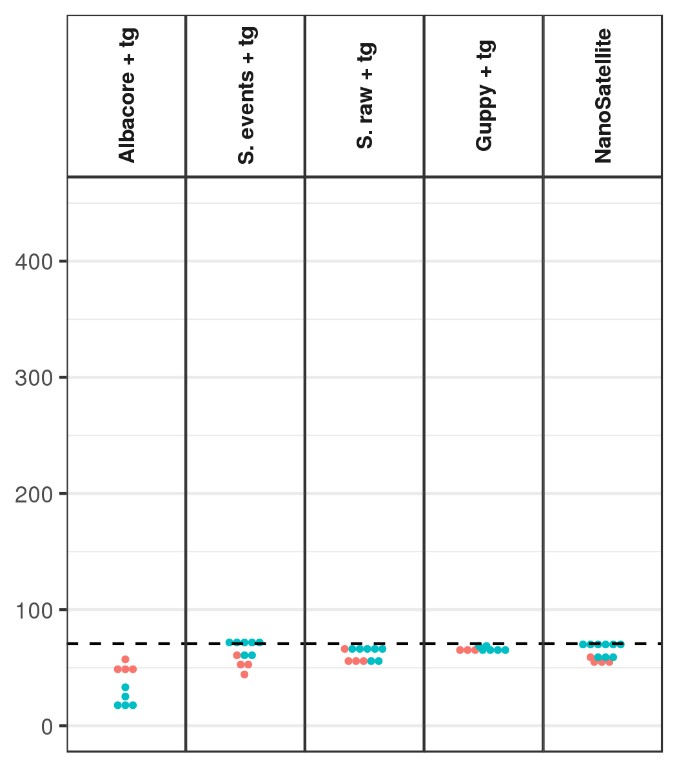 |
| --- | --- |
| **c) Subject03**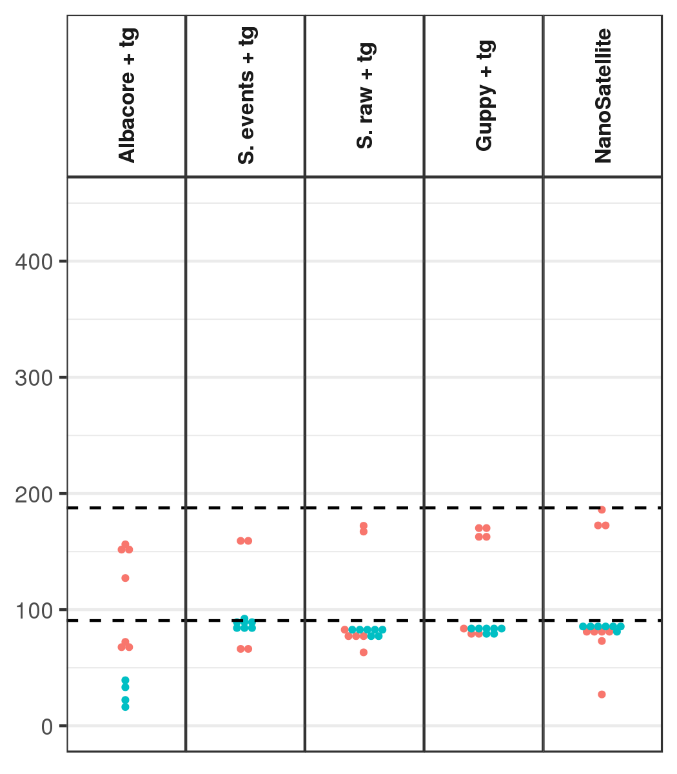 | **d) Subject04**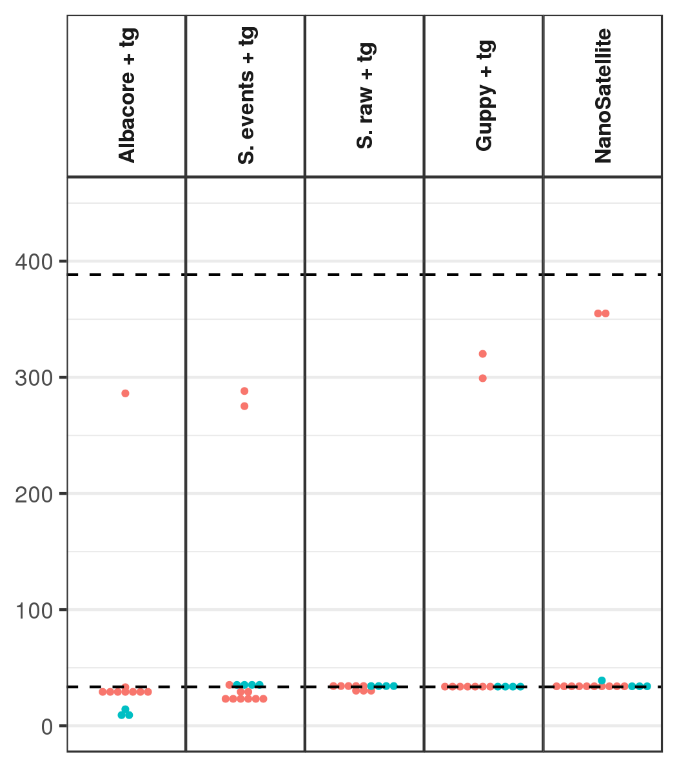 |
| **e) Subject06**  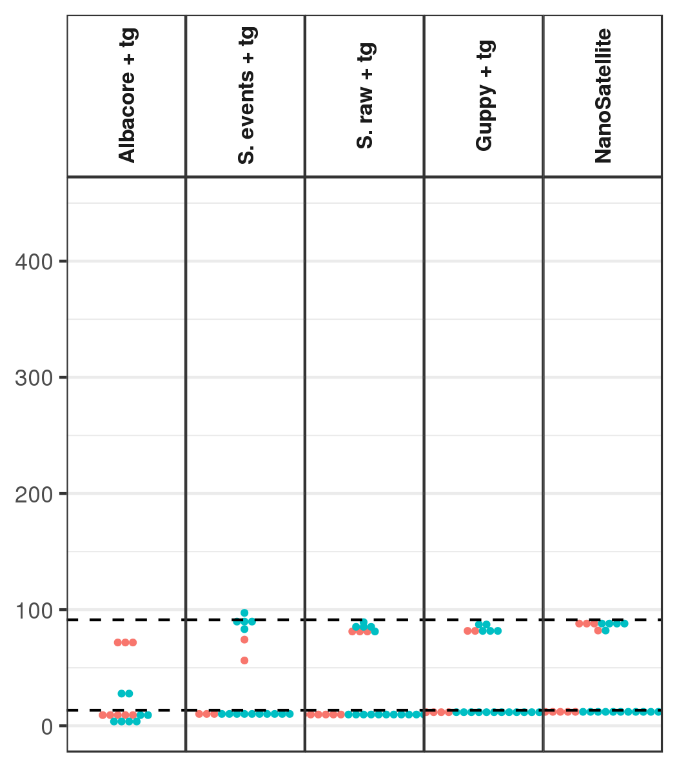 | **f) Subject07**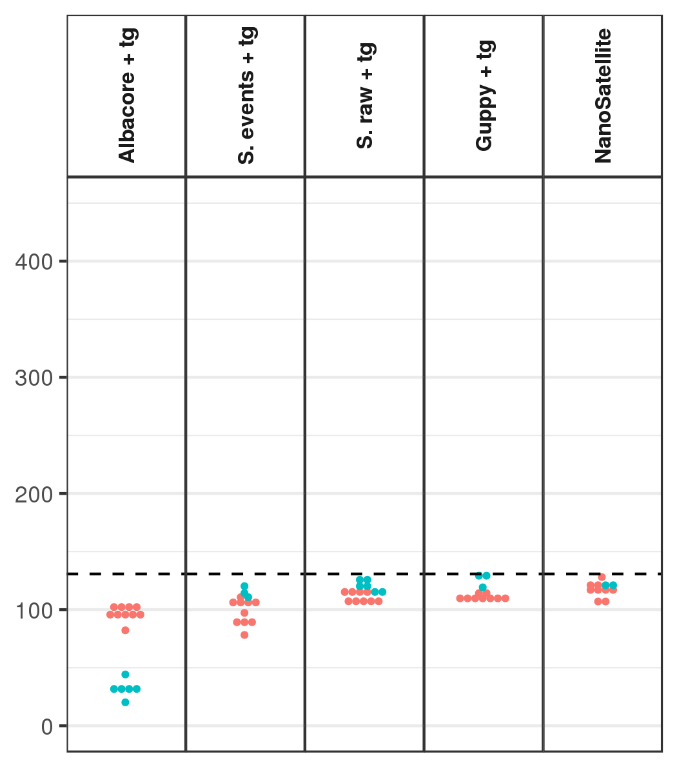 |
| **g) Subject08**  **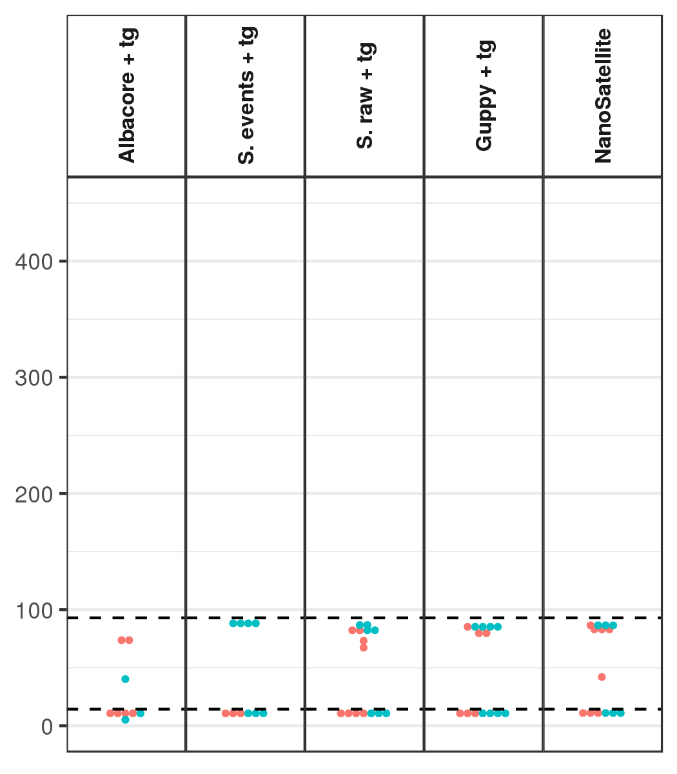** | **h) Subject09**  **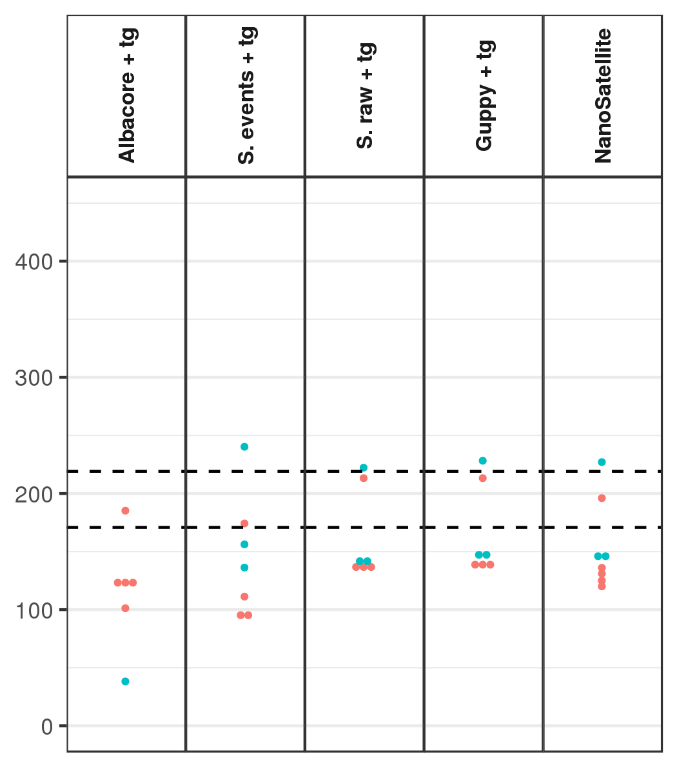** |
| **i) Subject10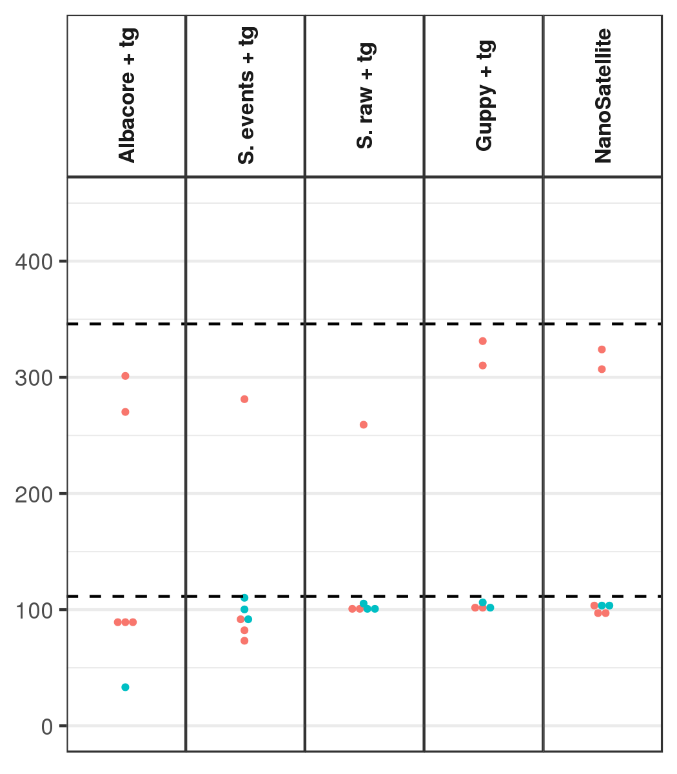** |  |

**Figure S5:** *ABCA7* VNTR TR length estimate comparisons of the Albacore + tandem-genotypes (tg), Scrappie (S.) events + tg, S. raw + tg, Guppy “flip-flop” base calling + tg, and the NanoSatellite approach, for all samples not yet included in Figure 2a and 2b. The number of tandem repeat units is depicted on the y-axis per positive strand (red) or negative strand (blue) PromethION sequencing reads (dots), and Southern blotting lengths are represented by dashed lines.


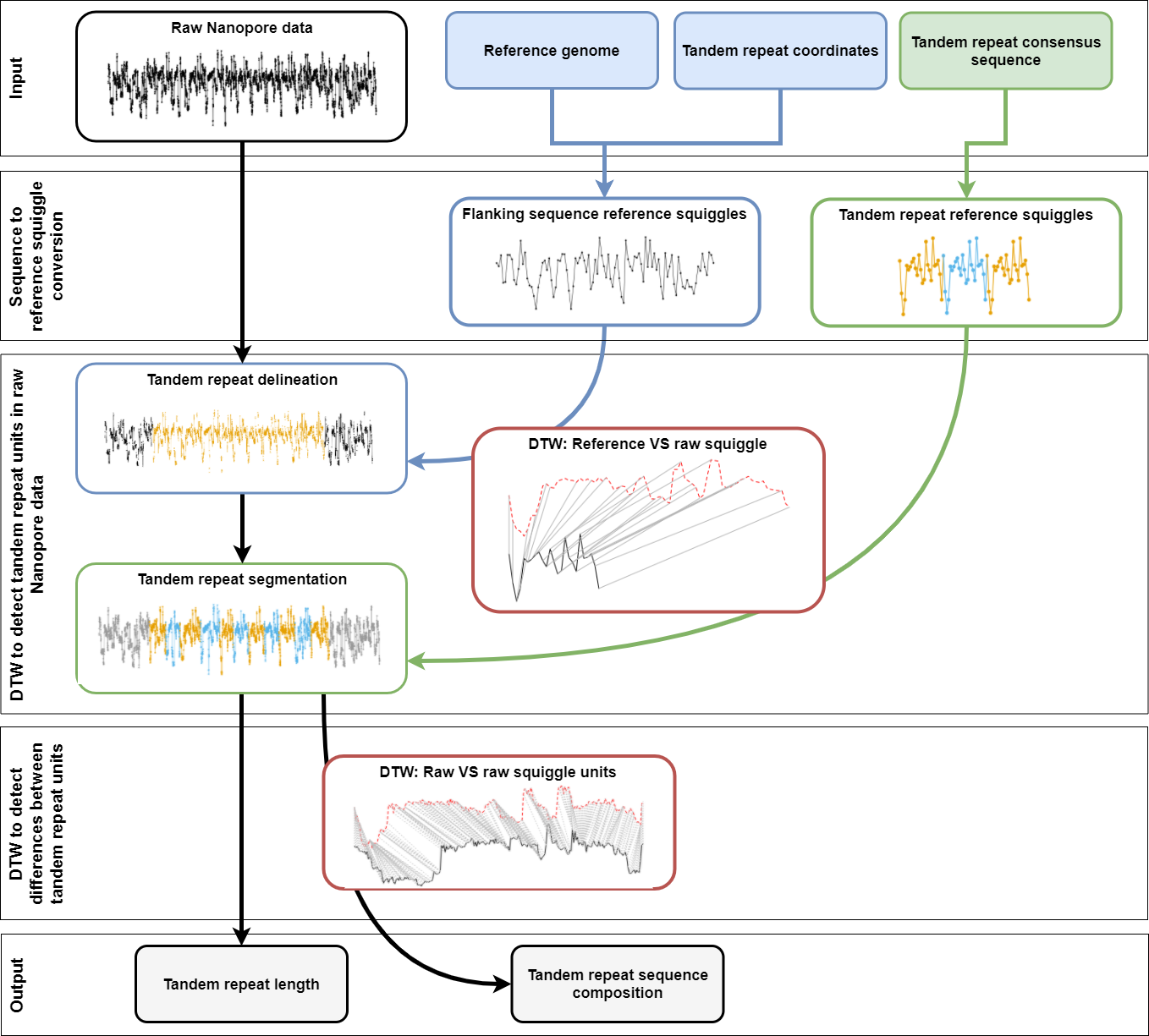


**Figure S6: A more detailed overview of the NanoSatellite method.** DTW = dynamic time warping


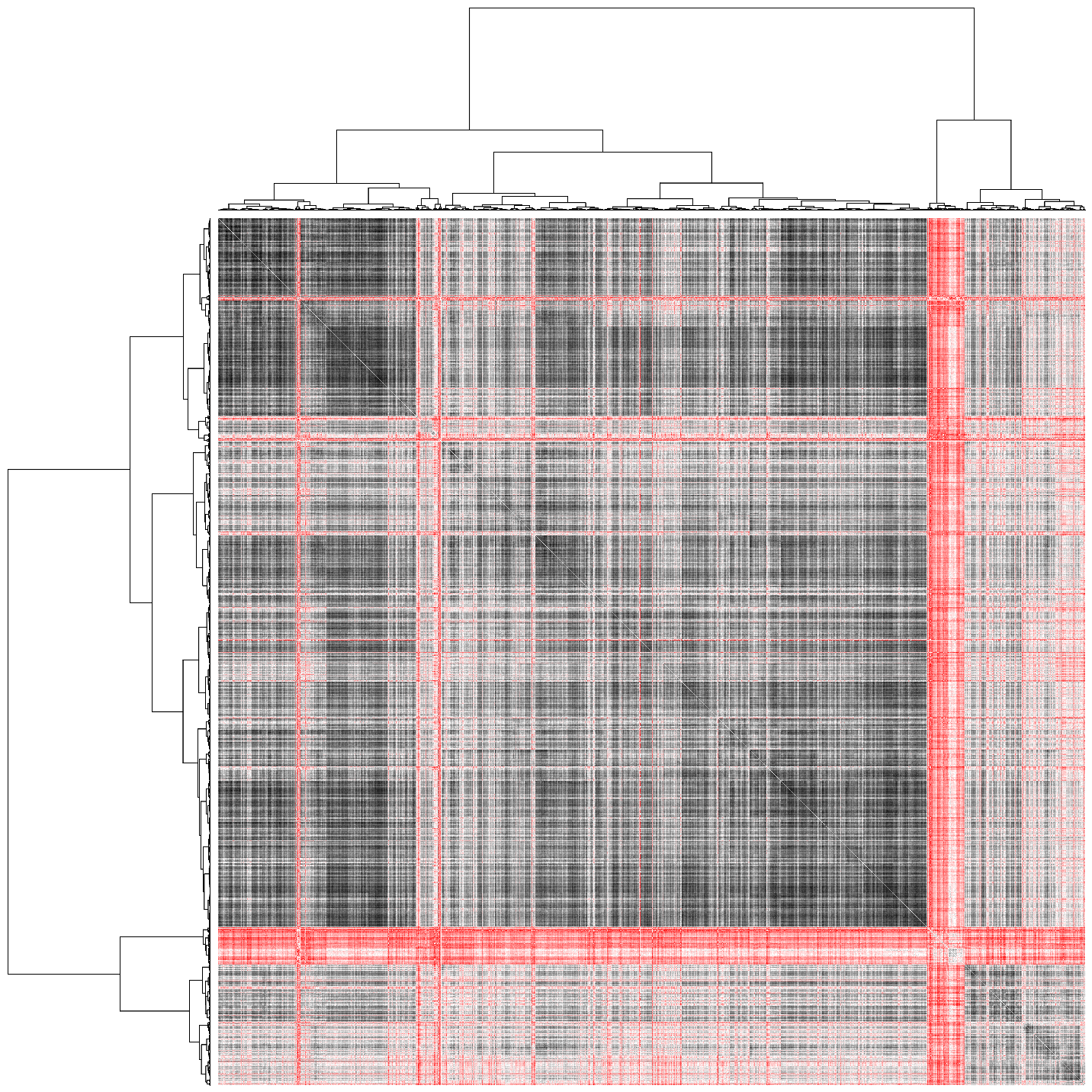


**Figure S7: Heatmap of positive strand *ABCA7* VNTR unit clustering.** DTW distances (as an inverse measure of sequence similarity) between the 11900 positive strand tandem repeat units are shown by a color scale: black corresponds to low distances (i.e. high sequence similarity between a pair of repeat units), white to medium distance, and red represents the highest distances (i.e. lowest sequence similarity between a pair of repeat units). Clustering was performed according to Ward’s method and represented in a dendrogram above and left of the heatmap. We created two clusters, (by cutting the dendrogram at the first two branches) and compared the corresponding squiggle centroid to reference squiggles of alternative VNTR motifs. The smallest cluster (bottom right) corresponds to a guanine insertion, or cytosine to adenine substitution at nucleotide ten of the *ABCA7* VNTR consensus motif.


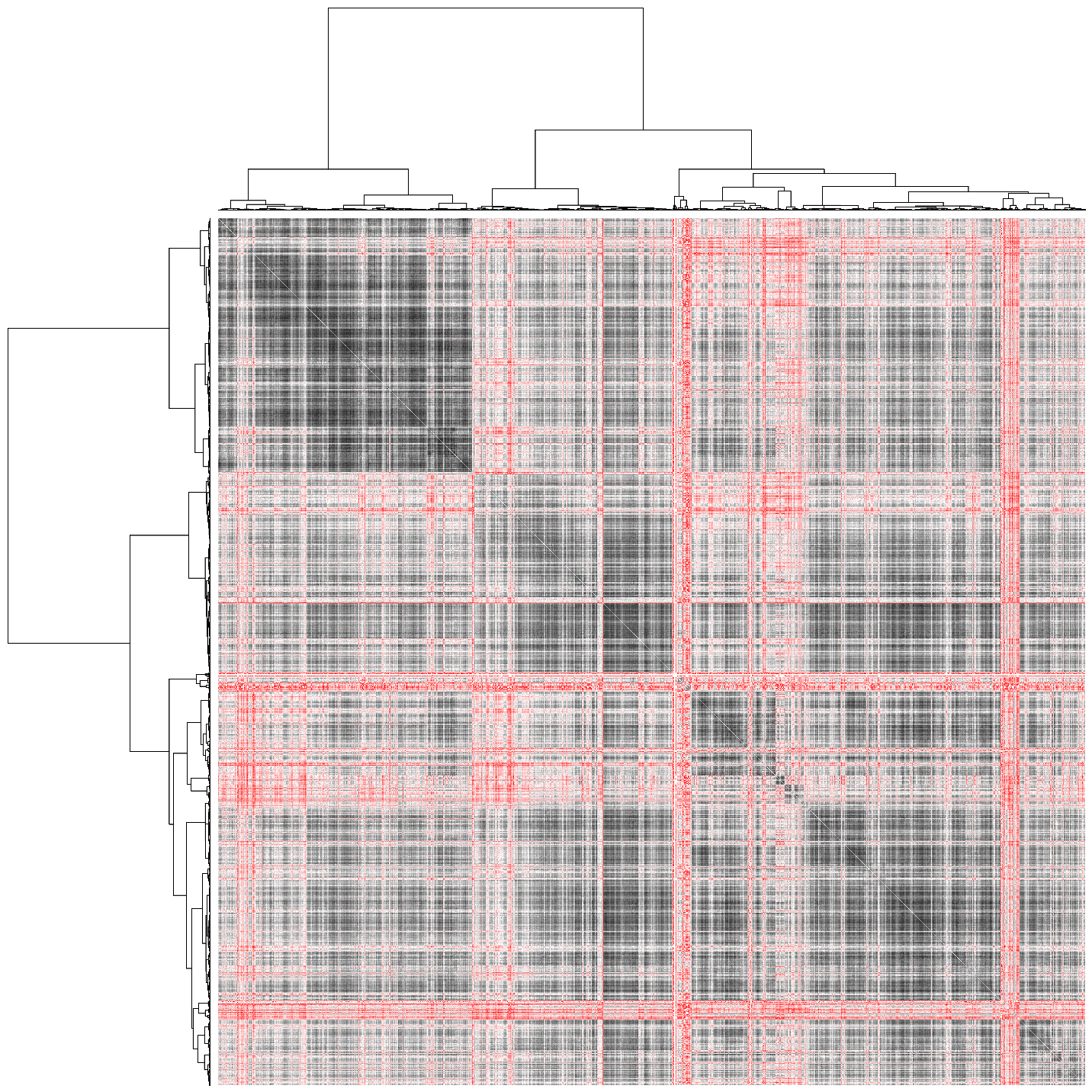


**Figure S8: Heatmap of negative strand *ABCA7* VNTR unit clustering.** DTW distances (as an inverse measure of sequence similarity) between the 7581 negative strand tandem repeat units are shown by a color scale: black corresponds to low distances (i.e. high sequence similarity between a pair of repeat units), white to medium distance, and red represents the highest distances (i.e. lowest sequence similarity between a pair of repeat units). Clustering was performed according to Ward’s method and represented in a dendrogram above and left of the heatmap. We created two clusters, (by cutting the dendrogram at the first two branches) and compared the corresponding squiggle centroid to reference squiggles of alternative VNTR motifs. The smallest cluster (top left) corresponds to a guanine insertion, or cytosine to a cytosine to thymidine substitution at nucleotide 21 of the negative *ABCA7* VNTR consensus motif.

| **a** | 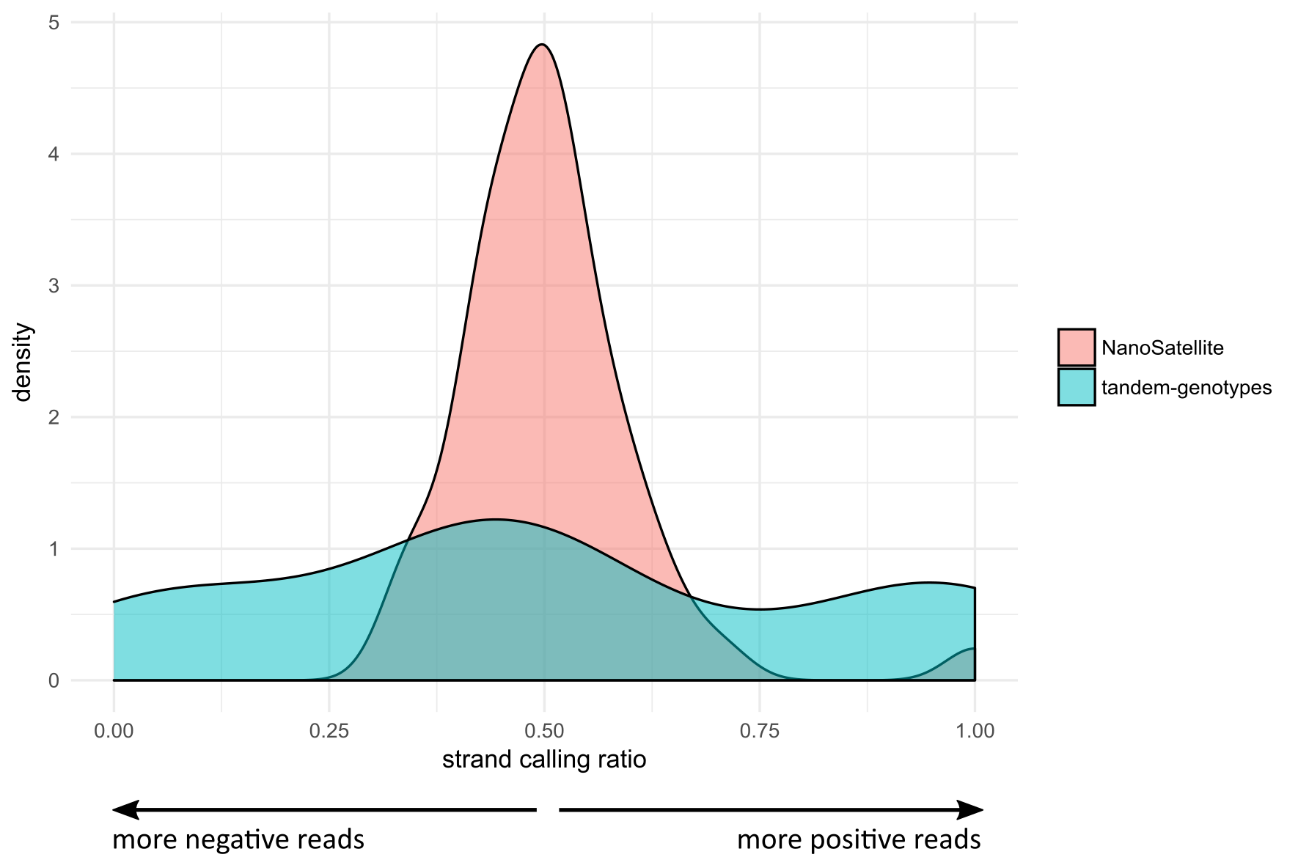 |
| --- | --- |
| **b** | 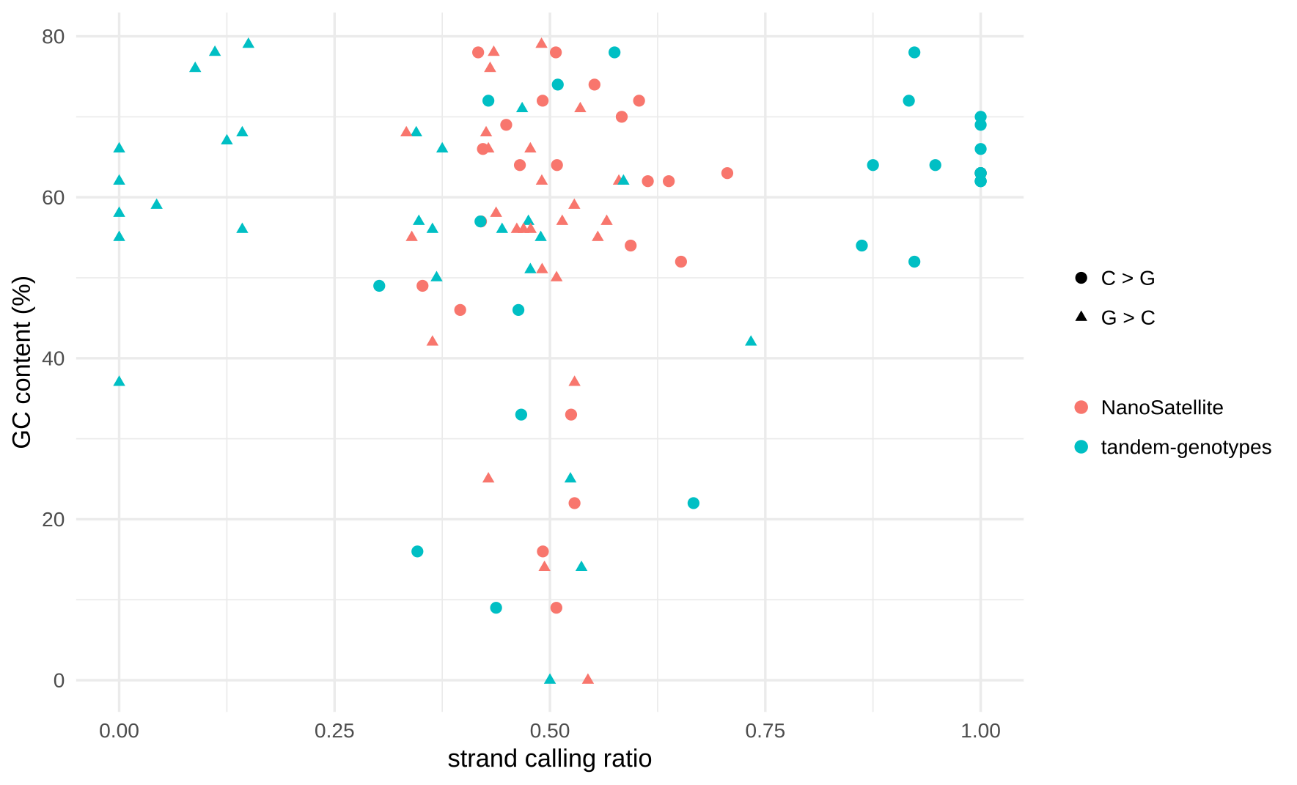 |

**Figure S9: Genome-wide tandem repeats: Comparison of strand-based output of tandem-genotypes and NanoSatellite.** The **s**trand calling ratio (x-axis) corresponds to the number of sequencing reads originating from the positive DNA strand, divided by the total number of reads. **(a)** The corresponding density distribution summarizing the 50 tested TRs, is shown for NanoSatellite (red) and Albacore + tandem-genotypes (blue). **(b)** Detailed overview of the effect of GC-content on the strand calling ratio. Each point corresponds to one TR, indicating whether the positive strand TR motif contains more cytosines than guanines (“C > G”, dots) or not (“G > C”, triangles).

| 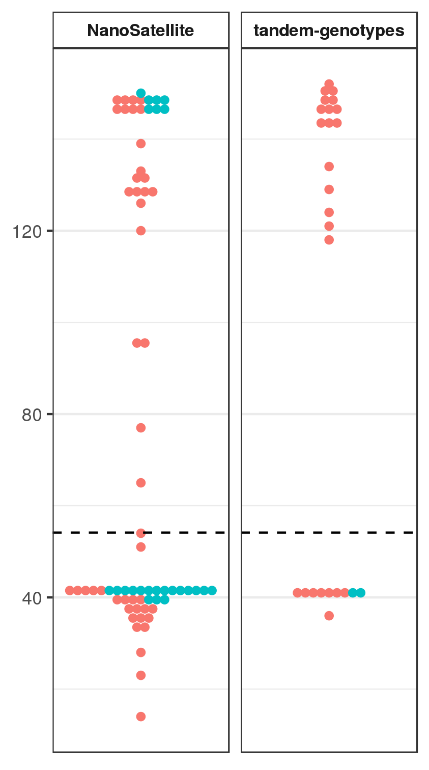 | 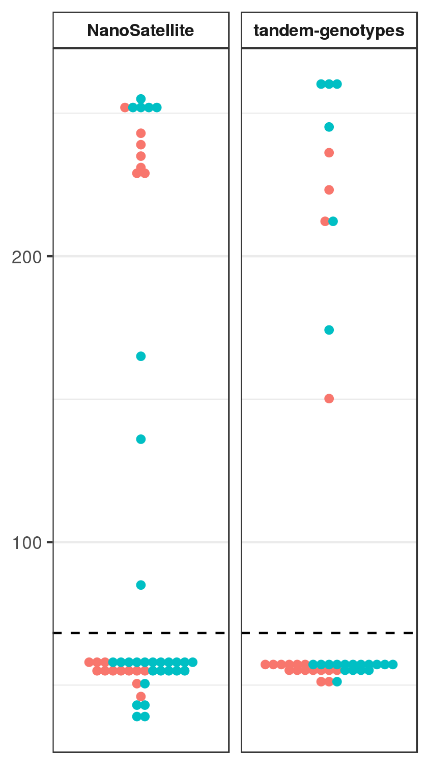 | 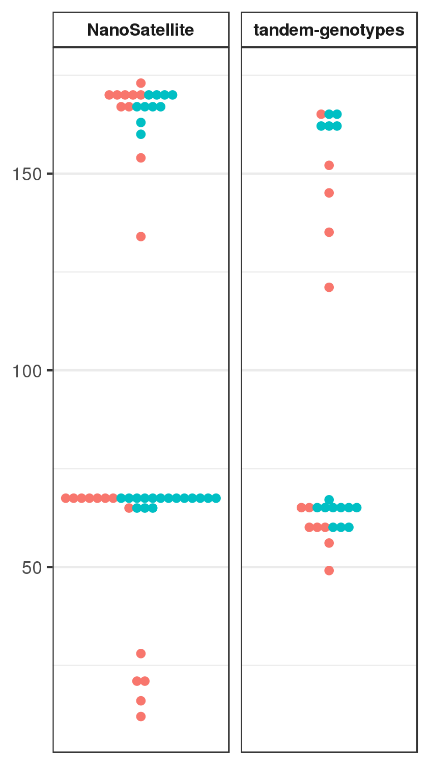 |
| --- | --- | --- |
| chr5:2145397-2147172  ACGACCTAATCACTTCCAGA TCCTCCACCTCCTCACCATC | chr5:14634207-14635661  AAACCACCACAGCACA CACACCCCTATGTAAT | chr6:6261813-6262518  CCCTCCAGGTGATTCTGAT GTGCTCCAAGCGTGGTGA |
| 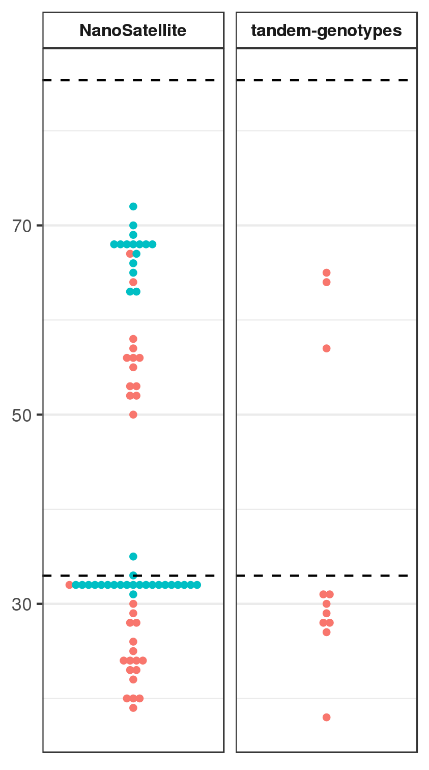 | 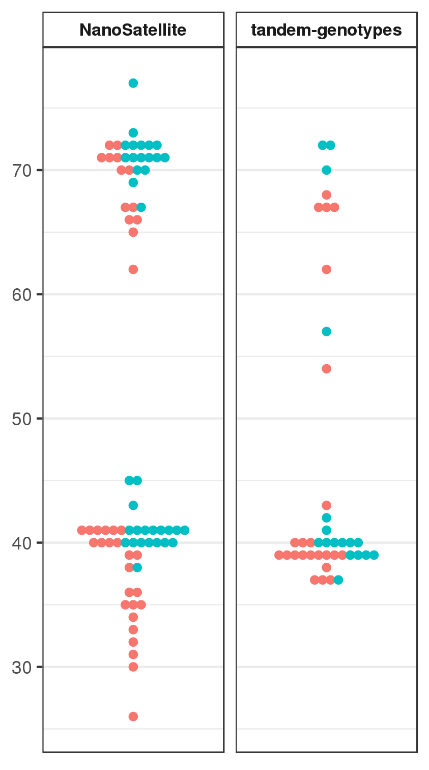 | 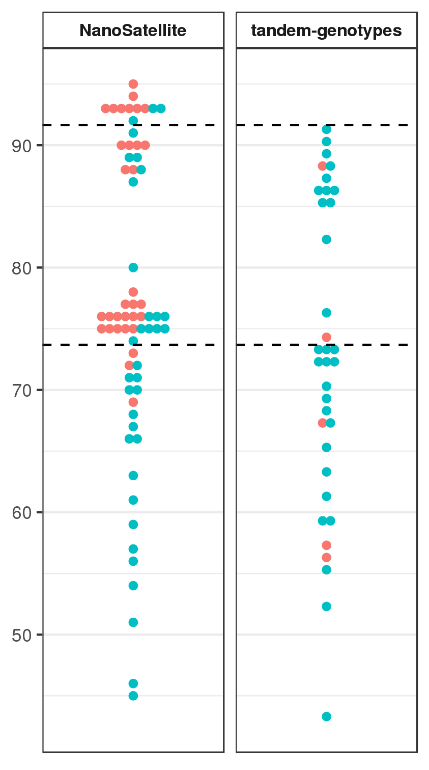 |
| chr7:401291-401903  CTCCCACTCACGCCTCAG CCTCCCTGTCCCGACTCC | chr7:675568-676096  CCGCGCCCTCAC GTCCTCCACCGG | chr9:140610468-140611021  GTGGTGGTGTCATGG TGGGGGAGGAAGTT |
| 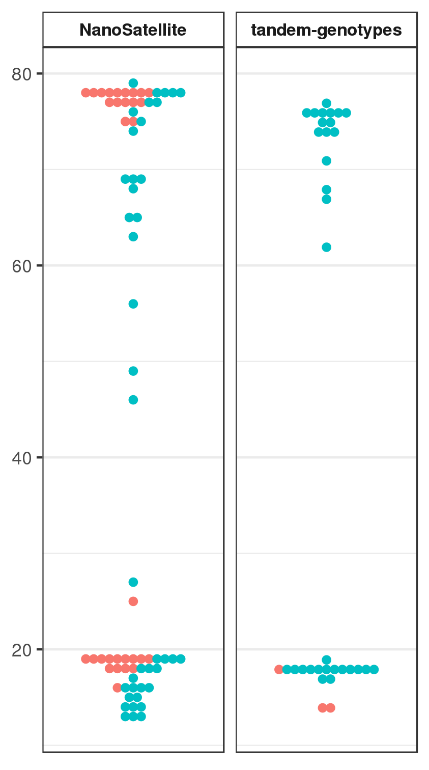 | 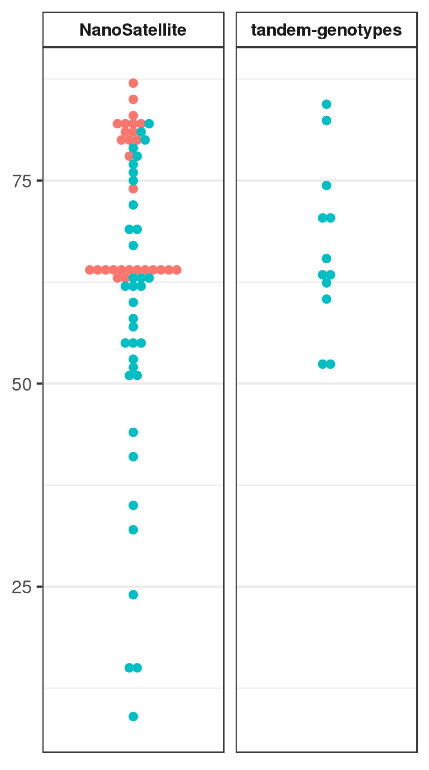 | 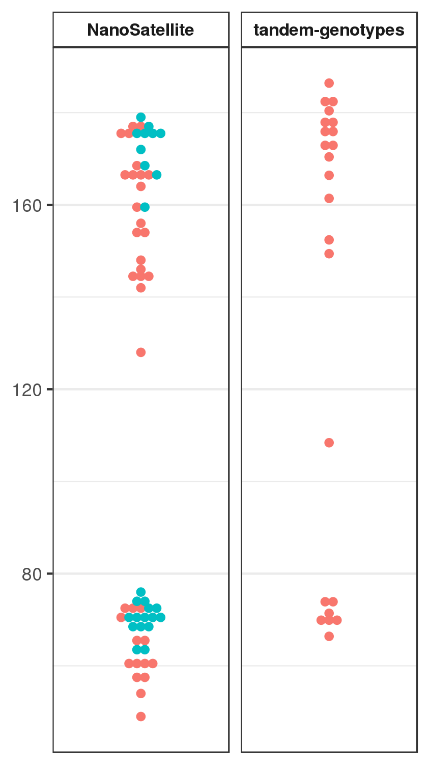 |
| chr11:1531224-1531893  GGAGGTCAGAGCCAGG GCTGCGGGGGGACCCG | chr11:65738209-65739132  GGATGGTGGGATTCA TGAGCTGAGGAGGG | chr13:114025714-114026238  CCCCTCCCTCCTCCTGAGAC |
| 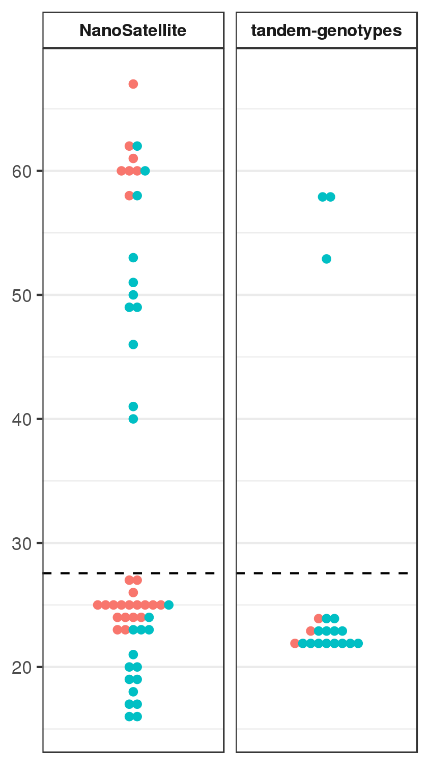 | 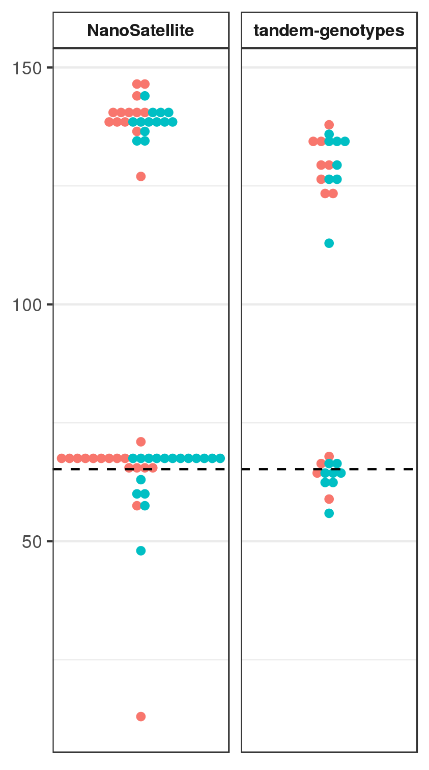 | 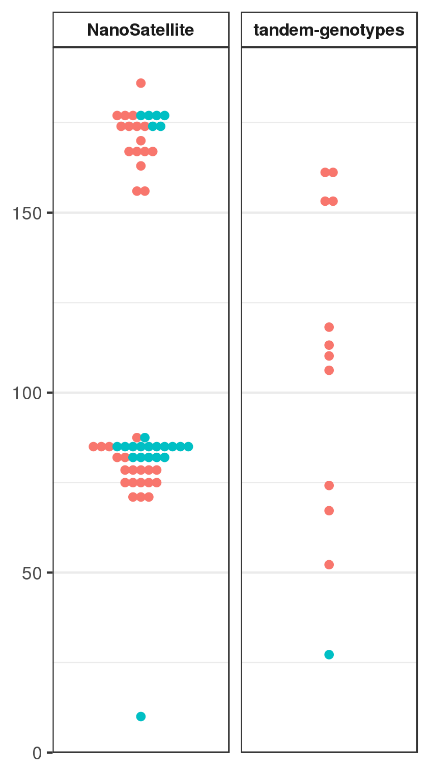 |
| chr17:81026020-81026769  GGAGGCGACAGCGGGGG CGACAACGGGGGTCCCG | chr19:410763-411353  TCCTGGGGGTCTGTCCACC GCCCCTGCTGGGCCCGGA | chr19:1049436-1050028  GTGAGCCCCCCAC CACTCCCTCCCC |
| 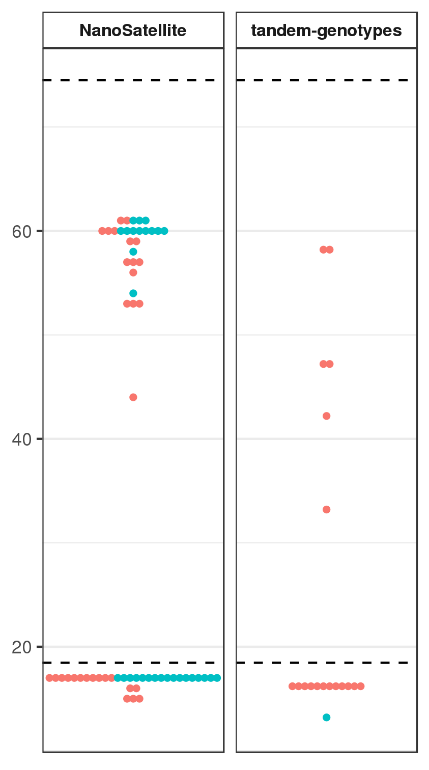 | 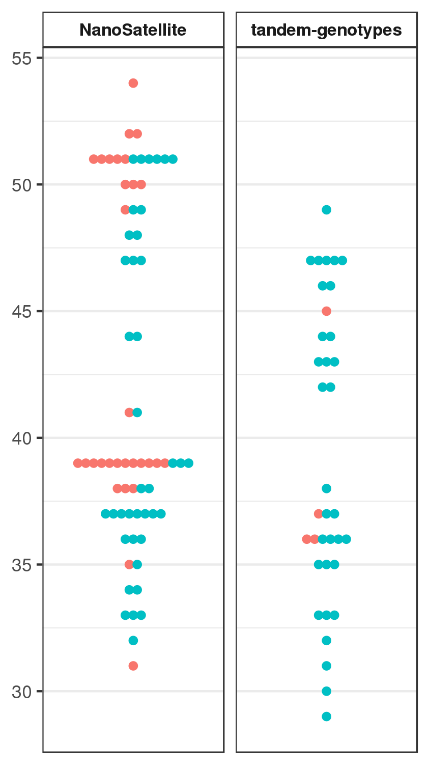 |  |
| chr19:1148463-1149068  CCCTTCCCACCCTCCTTCA CCCCAGAAAACCTCCCGC | chr20:622510-623079  GTTGCCTGGGGGGGGG GCCCAGCGGGGGTGGA |  |

**Figure S10: Genome-wide tandem repeats: comparison of NanoSatellite and tandem-genotypes for tandem repeats with preferred NanoSatellite length calling.** Each tandem repeat is represented by a NanoSatellite and Albacore + tandem-genotypes panel, the respective genomic region (hg19 coordinates), and the consensus motif of the tandem repeat. The number of tandem repeat units is depicted on the y-axis per PromethION sequencing read (dots) originating from the positive (red) or negative DNA strand (blue). Dashed lines correspond to expected TR sizes derived from PCR amplification of the TR region with DNA from NA19240 as the template, followed by gel electrophoresis. As these TRs are often made up by extreme nucleotide content and can assume relatively large sizes, not all TRs or alleles could be amplified and analyzed with confidence.

| **a** | 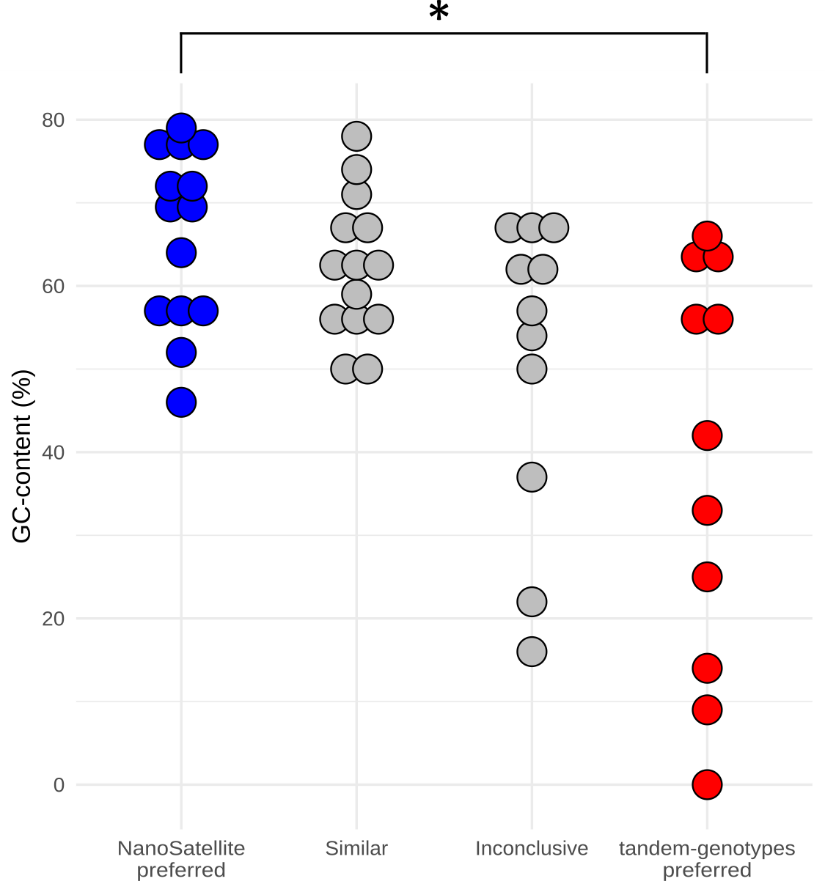 |
| --- | --- |
| **b** | 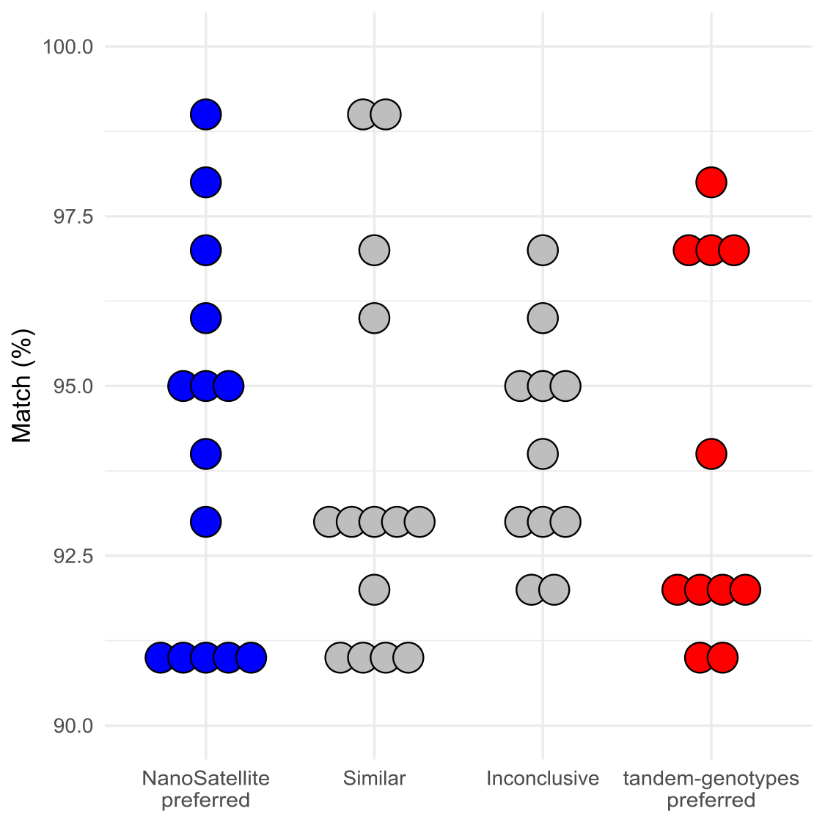 |
| **c** | 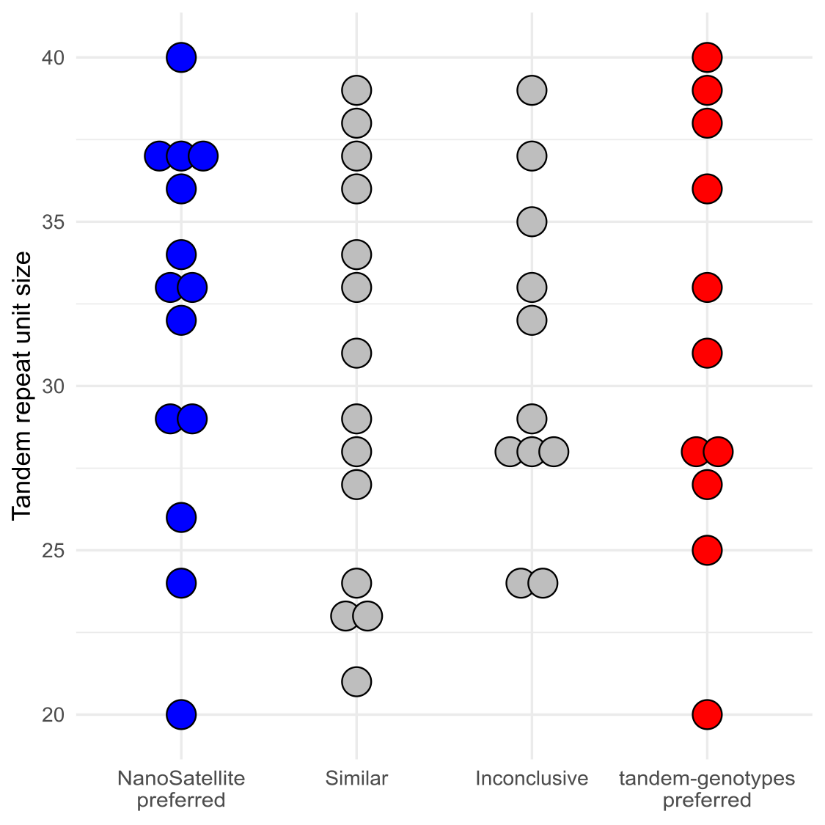 |
| **d** | 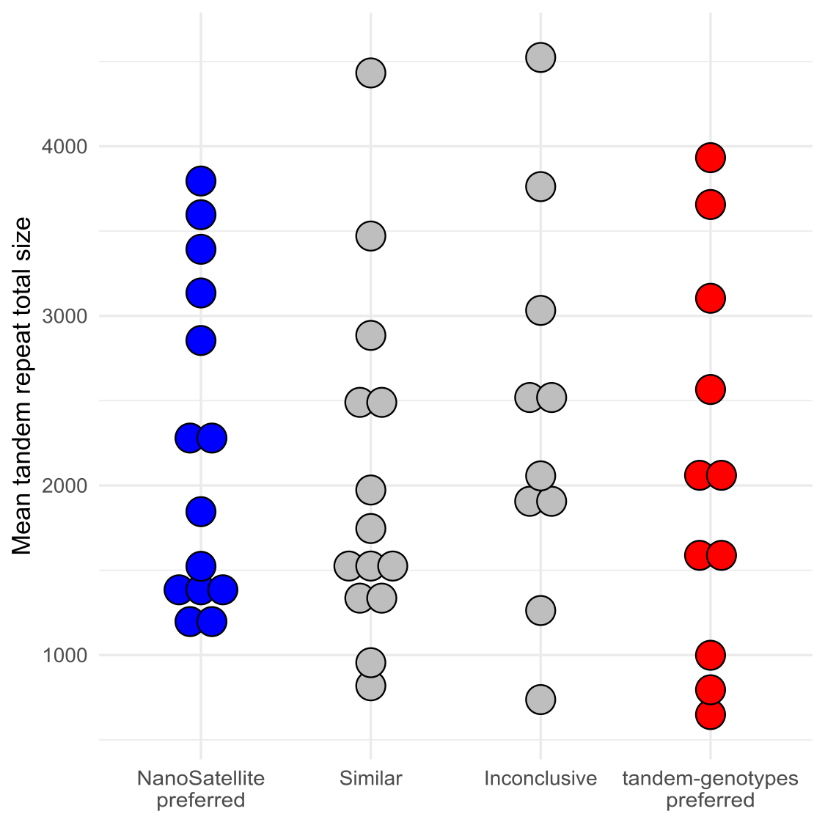 |

**Figure S11: Genome-wide tandem repeats: effects of tandem repeat features on NanoSatellite and Albacore + tandem-genotypes length estimations:** Lengths were estimated for the 50 cross-evaluated tandem repeats in NA19240 PromethION sequencing data. For some tandem repeats, both tools provided roughly comparable length estimates (“similar”), while others received different calls. For the latter, when one of the tools provided more informative length estimations, they were classified accordingly (“preferred” categories), if not they were regarded as “inconclusive”. **(a)** GC-content is compared between categories. **(b)** Comparison of the overall sequence match of the tandem repeat to the repeat unit consensus. **(c)** Comparison of tandem repeat unit size. **(d)** The mean TR size of NanoSatellite length calls in NA19240. * Significant difference (t-test p-value = 0.004).

| **Basecaller** | **Size** | **Match (%)** | **Indel (%)** | **A (%)** | **C (%)** | **G (%)** | **T (%)** |
| --- | --- | --- | --- | --- | --- | --- | --- |
| **Positive strand** |  |  |  |  |  |  |  |
| *Reference* | *26.0* | *93* | *2* | *12* | *61* | *11* | *13* |
| Albacore | 21.4 | 56 | 20 | 16 | 39 | 17 | 27 |
| Scrappie Events | 21.4 | 61 | 19 | 13 | 53 | 16 | 16 |
| Scrappie Raw | 23.4 | 65 | 17 | 12 | 59 | 14 | 13 |
| Guppy “flip-flop” | 22.6 | 65 | 17 | 14 | 55 | 14 | 15 |
| **Negative strand** |  |  |  |  |  |  |  |
| *Reference* | *25.0* | *93* | *2* | *13* | *11* | *61* | *12* |
| Albacore | 23.5 | 57 | 19 | 34 | 25 | 20 | 19 |
| Scrappie Events | 25.0 | 60 | 19 | 15 | 12 | 57 | 14 |
| Scrappie Raw | 24.1 | 64 | 18 | 15 | 12 | 57 | 14 |
| Guppy “flip-flop” | 24.7 | 64 | 17 | 16 | 15 | 53 | 15 |

**Table S1: Conventional nucleotide sequence summary statistics of the *ABCA7* VNTR.** Tandem Repeats Finder (TRF) was applied to all base called VNTR spanning reads. The average metrics of all patterns per base caller and strand direction are shown here compared to the *ABCA7* VNTR TRF pattern in the human reference genome. Size = number of nucleotides in the consensus repeat pattern. The following columns correspond to the percentage of nucleotides matching the consensus motif, percentage insertions or deletions (Indel) compared to the consensus, and compositions of adenine (A), cytosine(C), guanine (G), and thymine (T) of the TR.

| **Individual** | **Allele** | **Albacore** | **Scrappie events** | **Scrappie raw** | **Guppy** | **NanoSatellite** |
| --- | --- | --- | --- | --- | --- | --- |
| NA19240 | Smallest | 29 | 31 | 30 | 43 | 32 |
| NA19240 | Largest | 18 | 27 | 26 | 27 | 25 |
| Subject01 | Smallest | 4 | 8 | 5 | 3 | 8 |
| Subject01 | Largest | 6 | 2 | 1 | 1 | 2 |
| Subject02 | Homozygous | 9 | 11 | 11 | 9 | 13 |
| Subject03 | Smallest | 7 | 9 | 12 | 10 | 13 |
| Subject03 | Largest | 4 | 2 | 2 | 4 | 3 |
| Subject04 | Smallest | 11 | 13 | 12 | 11 | 14 |
| Subject04 | Largest | 1 | 2 | 0 | 2 | 2 |
| Subject05 | Smallest | 10 | 11 | 13 | 2 | 13 |
| Subject05 | Largest | 2 | 4 | 1 | 2 | 4 |
| Subject06 | Smallest | 13 | 13 | 17 | 16 | 16 |
| Subject06 | Largest | 3 | 7 | 8 | 7 | 9 |
| Subject07 | Homozygous | 16 | 13 | 15 | 12 | 11 |
| Subject08 | Smallest | 6 | 6 | 7 | 7 | 7 |
| Subject08 | Largest | 3 | 4 | 8 | 7 | 7 |
| Subject09 | Smallest | 5 | 6 | 5 | 5 | 6 |
| Subject09 | Largest | 1 | 1 | 2 | 2 | 2 |
| Subject10 | Smallest | 4 | 6 | 5 | 4 | 5 |
| Subject10 | Largest | 2 | 1 | 1 | 2 | 2 |

**Table S2: *ABCA7* VNTR spanning reads.** The number of sequencing reads attributing to an *ABCA7* VNTR length estimate is shown per individual, allele, and the TR calling method (base caller + tandem-genotypes pipeline or NanoSatellite).

| **Coordinates (hg19)** | **Tandem repeat unit sequence** | **Category** |
| --- | --- | --- |
| chr1:1317610-1318281 | AGAGGTCACCACCCCTTCCCAACAATCCAGTAACAATCC | Similar |
| chr2:7733927-7734388 | ATATAAATATATTTAATATAAATATATAAAAT | tandem-genotypes preferred |
| chr2:10676285-10676879 | TCGGGGGGTCCTGTGTATTGGGGTGTT | Similar |
| chr2:241565037-241565925 | GGGGTCTCAGAGTGGGGTGAGGCTGTGAT | Inconclusive |
| chr2:242529363-242530683 | TGATGATGATGGGCACTAGGACACTCAGAGCTGCTCGGC | tandem-genotypes preferred |
| chr3:175949-177324 | ACTGGATATAGTATTAAACACTGTATAT | tandem-genotypes preferred |
| chr3:195439614-195441312 | GGTCCTCCTGCCCTAACTCCTCCCTGACC | Similar |
| chr4:183752962-183753565 | CCTTCTCCTCTCCCCTCCTTCTCCTCTCCCC | Similar |
| chr4:190767165-190768372 | TCCGTGCCGTGTCCCTCGGCTCTCTCCCATTC | Inconclusive |
| chr5:309681-310163 | GTCCCCGTGCACCCCAGTGATGGCCGCC | Similar |
| chr5:1098174-1098929 | CTCTAACCCTCTGCACACCCAGCCCCC | tandem-genotypes preferred |
| chr5:2145397-2147172 | ACGACCTAATCACTTCCAGATCCTCCACCTCCTCACCATC | NanoSatellite preferred |
| chr5:14634207-14635661 | AAACCACCACAGCACACACACCCCTATGTAAT | NanoSatellite preferred |
| chr5:27426563-27427001 | ATAATGTACCTACATTATATCCGTCGAC | tandem-genotypes preferred |
| chr6:371049-372456 | AGCAGCAGTTGCAGTAGCTGTGGCAGGAGGAGTAGCAGC | Inconclusive |
| chr6:6261813-6262518 | CCCTCCAGGTGATTCTGATGTGCTCCAAGCGTGGTGA | NanoSatellite preferred |
| chr6:160590066-160590748 | ACATTTATTGTGTATAAATGTTATTTATACAT | tandem-genotypes preferred |
| chr6:168397815-168398216 | GGGGGCGGGGGAATCTGCAGAGATG | tandem-genotypes preferred |
| chr6:170803947-170805970 | GGGTTGGAGGAACTACAGAGCGGTGGTGAAGAGGAGGATG | tandem-genotypes preferred |
| chr7:401291-401903 | CTCCCACTCACGCCTCAGCCTCCCTGTCCCGACTCC | NanoSatellite preferred |
| chr7:675568-676096 | CCGCGCCCTCACGTCCTCCACCGG | NanoSatellite preferred |
| chr7:71144871-71145640 | TCCCTCCCTCCCTCCCTCCC | tandem-genotypes preferred |
| chr7:158707726-158710006 | TTGTGAAACGTCGACACGCTGGTTGACATTAAGGATGA | tandem-genotypes preferred |
| chr8:144153595-144155435 | GTTCCTGTTTGAGGAGACAGCCGTCGTCCTCGGG | Similar |
| chr9:140610468-140611021 | GTGGTGGTGTCATGGTGGGGGAGGAAGTT | NanoSatellite preferred |
| chr11:585208-585670 | GCCCTTTCCAGCTTGAGGTAGTA | Similar |
| chr11:980013-980572 | TGTCCTGGAGCGGTGACAGGCTGCCCGGTGCCG | Similar |
| chr11:1531224-1531893 | GGAGGTCAGAGCCAGGGCTGCGGGGGGACCCG | NanoSatellite preferred |
| chr11:65738209-65739132 | GGATGGTGGGATTCATGAGCTGAGGAGGG | NanoSatellite preferred |
| chr11:121055969-121056912 | TAGGGAAAGCAGATGTTCAATGCTTAATGAATGAG | Inconclusive |
| chr11:128737951-128738606 | ATTATGTATTATATTCTAATATATTATATAACATAT | tandem-genotypes preferred |
| chr12:131877044-131878391 | GTGGTGTGATGTGGAGGGTGGGT | Similar |
| chr13:112170232-112170671 | CCCCCCGGCTCCCTCACCTGC | Similar |
| chr13:114025714-114026238 | CCCCTCCCTCCTCCTGAGAC | NanoSatellite preferred |
| chr15:26002963-26004083 | CACCTGCTCCACCCTAACTCATTCCGAC | Inconclusive |
| chr15:29355102-29355885 | GTGGGGAGGGGAGTTGATCTGGTCAGTGTCTGTATGG | Similar |
| chr16:1002750-1004060 | CCAGCCTCCTACACGTCCACACAGACACCCACCCCAAA | Similar |
| chr16:1075242-1075713 | GGGTGCTGAGGTCCCCGGCTTGGA | Similar |
| chr16:88613258-88614296 | ACAGAGGGTGGGGAAGGCGAGGGG | Inconclusive |
| chr17:122702-123430 | GAGACCCCAGCGAGGGTGACGTCAGGGA | Inconclusive |
| chr17:81026020-81026769 | GGAGGCGACAGCGGGGGCGACAACGGGGGTCCCG | NanoSatellite preferred |
| chr18:73951886-73952321 | ATATGTATATATGTATACACACAC | Inconclusive |
| chr19:410763-411353 | TCCTGGGGGTCTGTCCACCGCCCCTGCTGGGCCCGGA | NanoSatellite preferred |
| chr19:1049436-1050028 | GTGAGCCCCCCACCACTCCCTCCCC | NanoSatellite preferred |
| chr19:1148463-1149068 | CCCTTCCCACCCTCCTTCACCCCAGAAAACCTCCCGC | NanoSatellite preferred |
| chr20:622510-623079 | GTTGCCTGGGGGGGGGGCCCAGCGGGGGTGGA | NanoSatellite preferred |
| chr20:45417476-45418119 | AGGTGTGTGTTTATTGGATGTGAGCGTGTGGGTGTGC | Inconclusive |
| chr21:46447954-46448733 | CTGGGGGTCACAGCGTCCTGCGGAGGGACCGTCTGT | Similar |
| chrX:8137158-8137951 | CTCCCTCCCCACACACCACCTCTTCCTCCCTCC | Inconclusive |
| chrX:28248096-28248584 | ATATCTATACATATAATACAGACATTAT | Inconclusive |

**Table S3: 50 cross-evaluated genome-wide tandem repeats.** Lengths were estimated for the 50 cross-evaluated tandem repeats, listed in this table, in NA19240 PromethION sequencing data. For some tandem repeats, both tools provided roughly comparable length estimates (“similar”), while others received different calls. For the latter, when one of the tools provided more informative length estimations, they were classified accordingly (“preferred” categories), if not they were regarded as “inconclusive”.
