# Supplementary material for: NanoSatellite: accurate characterization of expanded tandem repeat length and sequence through whole genome long-read sequencing on PromethION
Source: Genome Biol. 2019 Nov 14;20:239. doi: 10.1186/s13059-019-1856-3 (PMC6857246; doi:10.1186/s13059-019-1856-3)
Supplement: Supplementary file 2 — Additional file 2. Review history. [file 13059_2019_1856_MOESM2_ESM.docx]

**1^st^ round**

**Reviewer 1**

The manuscript describes a novel approach to genotype VNTR using an alignment free squiggle based algorithm for Nanopore long-read sequencing data. Authors demonstrate the feasibility of long-read whole genome sequencing of human genomes using Nanopore PromethION and how squiggle data can be used to improve characterisation of tandem repeats. Although the approach is only applicable to Nanopore sequencing, it has the potential to improve TR analysis.

I have the following questions/comments to the authors,
1)     In line 110, authors mention about evaluating the data for nucleotide modification, however there was not any supporting data or results in the manuscript. If the authors are referring to base modifications, they should present evidence or remove this comment.
2)     Nanosatellite seems to perform better in GC rich TRs, does the authors have any reasoning as to why it is the case.  
3)     Authors present evidence for strand bias in ABCA7 VNTR, do authors notice strand bias in other TRs that are not GC rich? 
4)     Could the authors comment on computing speed for TR analysis using Nanosatellite. Is it efficient for genome-wide analysis of TRs.
5)     It is unclear why the authors have used k-means clustering for the detection of diploid TR alleles (line 500). Earlier they mentioned using hierarchical clustering of TR units (line 472). Could the authors please clarify?
6)     In the analysis of other TRs, authors classify TRs as "preferentially called with NanoSatellite and preferentially assessed with tandem-genotypes". It is unclear how the authors performed this classification, was any sizing results (PCR or southern blotting) used to confirm the TR lengths?
7)     In Figure S10, it is worth including another plot on comparison of the total length of the repeat region to assess the difference between categories.

Authors have presented a very useful approach for TR analysis. I hope authors find these comments useful to improve their manuscript.

**Reviewer 2**

In the manuscript "Accurate characterization of expanded tandem repeat length and sequence through whole genome long-read sequencing on PromethION", De Roeck et al. describe their work using the PromethION to sequence 10 human samples and a cell line, then exploring this data to measure VNTRs length and sequence. This is a great data set and is applied to an interesting problem, but I have concerns about the thoroughness of the data analysis as pertaining to VNTRs.  Specific points listed below.


1. The authors suggest that yield was affected by the number of pores available over time (Sup Fig 1) - but do not plot the number of active pores as it relates to yield.  This would be an interesting figure/plot to add.
2. I hate to do this, but it's probably a good idea to recall their data with the most recent guppy v3 basecaller (with the "flip-flop" addition) - accuracy has improved and it may help with the VNTR length estimation.
3. The authors - in Figure S3 - assert a high correlation between mean read length and average DNA length prior to library prep (side note, label the X-axis as something clearer like "average side of input DNA".  But Subject09, for example, seems to have an average fragment size of ~37.5kb, yet a mean read length of only ~17.5kb?  The authors do not discuss this discrepancy?
4. It would be useful to break Table 2 into a supplementary table listing the number of spanning reads per sample, as the authors note that only a few (1 for Subject05 and Subject 10 for example) spanning reads were present for some of the samples.
5. Based on the analysis done in figure 2 and figure S4 the authors do not address the issue with coverage. Some patient samples have as few as two reads spanning the length of the larger allele. One possible concern, especially examining Fig S5 is somatic variation of these repeats. Though a quick literature search did not reveal evidence of ABCA7 having known somatic variation, generally speaking, TRs can be unstable. The authors emphasize that this technique characterizes TRs with a single flow cell, and with their coverage they should be able to collect more reads which span the repeat. Is the coverage anomalously low in this area?  Is it it a function of the read length (i.e. does sample 9 have more reads which span ABCA7)?
6. Seems to be very little information on the actual operation of "NanoSatellite" beyond that it is a DTW based algorithm.  Similarly, what distances for the squiggle TR units were input into the unsupervised hierarchical clustering algorithm? I see Figure S6 says Ward distance from DTW distances, but it's still not clear to me what "DTW distance" means?  I can assume the authors mean the DTW based length of the VNTR?
7. Which 50 VNTRs were probed in NA19240? And what does a "preferential" call mean here?  
8. And what is the "truth" set for NA19240?  I understand that Southerns were used for ABCA7 - does such data exist for these other VNTRs in NA19240?
9. A bit more about a "truth" set - ideally this algorithm would be verified against known truth.  A plasmid with known amounts of synthetic repeats to verify the accuracy of the algorithm at the single molecule level is an important step that should be taken before other groups begin the implementation of NanoSatellite. 
10. Also - I note that the authors do not discuss the TRs where "preferential" calls are made by tandem-genotypes or similar with both tools.  This data suggests that NanoSatellite at these other VNTRs does not provide a significant advantage (based on numbers alone at least.  Examination of Fig S9 bears this out to some extent - not knowing the "truth" of these VNTRs makes this data difficult to interpret.
11. It would be beneficial to note some of the limitations of NanoSatellite. For example, the minimal length repetitive unit that can be accurately identified by NanoSatellite. 
12. Lastly, the authors need to determine what impacts the performance of NanoSatellite and why it performs better on certain TRs. Groups utilizing this software tool need to be able to assess if it will perform well for a specific TR. In order for that to be possible, there need to be specific guidelines for the structure of the repetitive unit that is most compatible with this algorithm. 
13. The authors say that there was "no clear relation between N50 read length and yield" - but this is clearly untrue as they state earlier that "lowest yield for the sequencing run with the longest DNA fragments".  The longer N50 sample, in that case, has a markedly lower yield, but at the same time not the high N50 one would expect.  I'm well aware that this is an ongoing problem with getting HMW DNA from extraction actually to the pore for sequencing, I just think this section needs more clarity.

**Authors’ response**

Reviewer 1

The manuscript describes a novel approach to genotype VNTR using an alignment free squiggle based algorithm for Nanopore long-read sequencing data. Authors demonstrate the feasibility of long-read whole genome sequencing of human genomes using Nanopore PromethION and how squiggle data can be used to improve characterisation of tandem repeats. Although the approach is only applicable to Nanopore sequencing, it has the potential to improve TR analysis. I have the following questions/comments to the authors, 1)In line 110, authors mention about evaluating the data for nucleotide modification, however there was not any supporting data or results in the manuscript. If the authors are referring to base modifications, they should present evidence or remove this comment.

Author reply: Upon the reviewer’s request, we no longer mention “nucleotide modifications” in the manuscript on line 95 (formerly line 110).

2)Nanosatellite seems to perform better in GC rich TRs, does the authors have any reasoning as to why it is the case.

Author reply: The main reason is most likely that conventional methods perform worse in GC-rich sequences, potentially due to an underrepresentation of these sequences in training data for base calling neural network software. This also leads to the observation of “strand-bias” in conventional methods, which is visualized in Figure S9 and as we describe in reply to your next comment, more particularly caused by a loss of guanine-rich sequencing reads, which we now also mention in the results on lines 219 – 220 and 230 – 231 of the manuscript.

3)Authors present evidence for strand bias in ABCA7 VNTR, do authors notice strand bias in other TRs that are not GC rich?

Author reply: To answer this question, we added panel “b” to Figure S9. This figure demonstrates that particularly the GC-rich sequences are affected by strand bias, and within this group, the direction of bias is affected by the distribution of cytosines and guanines in the two DNA strands. In particular, guanine-rich sequencing reads are underrepresented, which we also noted in the manuscript on line 219 – 220 and 230 – 231.

4)Could the authors comment on computing speed for TR analysis using Nanosatellite. Is it efficient for genome-wide analysis of TRs.

Author reply: Since NanoSatellite is based on a completely new paradigm, the algorithm is not as time-optimized as conventional variant calling tools, and we therefore do not recommend the use of NanoSatellite for genome-wide analysis. This is, however, not the main goal of NanoSatellite. We wanted to develop an algorithm that provides a solution to some of the problems with conventional TR length calling. For users that are interested in genome-wide TR analysis, we would recommend to first apply conventional tools (e.g. guppy “flip-flop” base calling followed by LAST alignment and tandem-genotypes) and follow up with NanoSatellite for those TRs which are not showing accurate results. We now reference to this in the abstract and in the discussion on line 359 – 363.

5)It is unclear why the authors have used k-means clustering for the detection of diploid TR alleles (line 500). Earlier they mentioned using hierarchical clustering of TR units (line 472). Could the authors please clarify?

Author reply: Both clustering methods are applied on different types of data and with a different purpose. k-means clustering with two centers is applied on TR lengths (i.e. the total number of tandem repeat units which is observed in one sequencing read). As such, all TR lengths are divided into two groups which should correspond to the two alleles of the TR of interest; which is the expected for most TRs under study. The top 50 polymorphic TRs were chosen, which reduces the odds for homozygous TR lengths. Furthermore, the TRs have relatively large repeat unit motifs (20-40bp), reducing the odds for three or more alleles within one individual due to somatic mutations. Hierarchical clustering was not used on tandem repeat length, but on the DTW distance between squiggles of tandem repeat units, with the results shown in Figure S7 and S8. As such, we aim to obtain an overview of the different nucleotide sequences between TR units, and subsequently any number of TR unit groups can be differentiated (in contrast to diploid TR lengths, TR unit sequences can have much more variance). To provide more clarity in the manuscript, we adapted the methods section on line 560 – 563.

6)In the analysis of other TRs, authors classify TRs as "preferentially called with NanoSatellite and preferentially assessed with tandem-genotypes". It is unclear how the authors performed this classification, was any sizing results (PCR or southern blotting) used to confirm the TR lengths?

Author reply: For many TRs, it is very difficult to obtain good, experimentally validated “truth” data sets. Southern blotting is too low throughput for a large number of TRs and PCR is often inadequate due to the difficult TR sequence compositions and long lengths. We attempted PCR validation of TR lengths and for those that were successful, we now added the gel electrophoresis determined lengths to Figure 5 and Figure S10. Nevertheless, we believe there is another way to assess the quality of TR length calls made by long-read sequencing: assessment of the degree to which length estimates from independent sequencing reads cluster together in diploid alleles, and whether length estimates from both DNA strands overlap. We see that when length estimates cluster tightly in two groups, they also correspond well to validated Southern blot or PCR lengths when available. The same is observed in the Genome Biology paper on the tandem-genotypes algorithm (Mitsuhashi et al., 2019, https://genomebiology.biomedcentral.com/articles/10.1186/s13059-019-1667-6). A preferential call is made when either of the tools shows tighter clustering of read lengths, or provides more evidence of two alleles. Figure 5 is an example of “NanoSatellite preferred” calls now partially validated by PCR, as explained below. NanoSatellite provides more evidence of a second, larger allele by showing tighter clustering of read lengths, and the presence of length estimations from negative DNA strands in the left panel. We added more information about the PCR validations and preferential calls in the methods on lines 560 – 563 and 565 – 573, in the legend of Figure 5, in the results on line 226 – 227 and in the discussion on line 327 – 345.

7)In Figure S10, it is worth including another plot on comparison of the total length of the repeat region to assess the difference between categories.

Author reply: We thank the reviewer for the suggestion, and added this plot to figure S11 (former figure S10), and adapted the methods section on line 564 – 565. No effect of total TR length was observed on tool performance.

Authors have presented a very useful approach for TR analysis. I hope authors find

these comments useful to improve their manuscript.

Reviewer 2

In the manuscript "Accurate characterization of expanded tandem repeat length and

sequence through whole genome long-read sequencing on PromethION", De Roeck et

al. describe their work using the PromethION to sequence 10 human samples and a

cell line, then exploring this data to measure VNTRs length and sequence. This is a

great data set and is applied to an interesting problem, but I have concerns about the

thoroughness of the data analysis as pertaining to VNTRs. Specific points listed

below.

1. The authors suggest that yield was affected by the number of pores available over time (Sup Fig 1) - but do not plot the number of active pores as it relates to yield. This would be an interesting figure/plot to add.

Author reply: Upon the reviewer’s request, we added a panel to Figure S1 with the number of active pores over time.

2. I hate to do this, but it's probably a good idea to recall their data with the most recent guppy v3 basecaller (with the "flip-flop" addition) - accuracy has improved and it may help with the VNTR length estimation.

Author reply: As the reviewer noted, “flip-flop” base calling is indeed a very recent development with a release to the general community on 26/2/2019 (Guppy v2.3.5) and, as far as we know, the first and only peer-reviewed mentioning of Guppy “flip-flop” base calling occurred on 24/6/2019 (Wick et al., 2019, https://genomebiology.biomedcentral.com/articles/10.1186/s13059-019-1727-y), after we performed our analyses and submitted this manuscript. Upon the reviewer’s request we now recalled our data with Guppy “flip-flop” v2.3.5 and repeated the downstream alignment, tandem-genotypes, statistics, and graphical representation steps. We observed that Guppy “flip-flop” base calling improves accuracy, which is also translated into better TR length estimations, achieving slightly better accuracy and precision than NanoSatellite for benign lengths of the ABCA7 VNTR (Table 2). Nevertheless, Guppy “flip-flop” is not perfect. First of all for the expanded diseasecausing alleles we observed that Guppy still loses reads, which is important information, since these expanded alleles are more difficult to cover. Secondly, expanded read calls are further from the Southern blotting lengths than predictions by NanoSatellite. Thirdly, we see that particularly indels still persist within the TR repeat motifs, which makes it difficult to assess alternative sequencing motifs through conventional methods, while NanoSatellite could resolve them. Furthermore, for variant calling in general, it usually pays off to use more than one tool to obtain more accurate calls. Since the NanoSatellite and tandem-genotypes algorithms are based on completely different paradigms, they can serve as a second opinion to each other. In this revised manuscript we recommend readers to first analyze TRs of interest using the conventional approach coupled with Guppy “flip-flop” base calling, and to apply NanoSatellite to TRs with uncertain calls. To reflect these changes, we adapted the abstract, the Results on lines 142, 154 and a shift in text from line 192 to 161, the Discussion on line 297 and 360, the Conclusion on line 374, the Methods on lines 444 and 456, Figure 2, Figure S5, Table 2, and Table S2.

3. The authors - in Figure S3 - assert a high correlation between mean read length and average DNA length prior to library prep (side note, label the X-axis as something clearer like "average side of input DNA". But Subject09, for example, seems to have an average fragment size of ~37.5kb, yet a mean read length of only ~17.5kb? The authors do not discuss this discrepancy?

Author reply: We adapted the x-axis title upon the reviewer’s request. The difference between both size measures can most likely be attributed to several factors. First, short fragments are more preferentially sequenced than long fragments, as the free DNA ends which need to make contact to the nanopore are more easily accessible in short fragments. Secondly, while we aim to avoid this by careful experimental handling, DNA can be sheared or nicked during library preparation. Thirdly, single strand nicks do not impact the dsDNA size as measured by Fragment analyzer and if they are not successfully repaired during library preparation, they will lead to shorter sequencing reads, since nanopores sequence single strand DNA. We now discuss this discrepancy in the manuscript on line 115 – 118.

4. It would be useful to break Table 2 into a supplementary table listing the number of spanning reads per sample, as the authors note that only a few (1 for Subject05 and Subject 10 for example) spanning reads were present for some of the samples.

Author reply: We now added Table S1 containing more details and a reference in the legend of Table 2 (Table S1 in the previous manuscript submission is renamed to S2).

5. Based on the analysis done in figure 2 and figure S4 the authors do not address the issue with coverage. Some patient samples have as few as two reads spanning the length of the larger allele. One possible concern, especially examining Fig S5 is somatic variation of these repeats. Though a quick literature search did not reveal evidence of ABCA7 having known somatic variation, generally speaking, TRs can be unstable. The authors emphasize that this technique characterizes TRs with a single flow cell, and with their coverage they should be able to collect more reads which span the repeat. Is the coverage anomalously low in this area? Is it it a function of the read length (i.e. does sample 9 have more reads which span ABCA7)?

Author reply: First, total yield of the sequencing run has the largest effect on coverage for the most conventional ABCA7 VNTR lengths (e.g. while Subject09 has the largest read lengths, this sequencing run also had the lowest overall sequencing yield (Figure S1) and therefore, Subject09 has a relatively low coverage / number of spanning ABCA7 VNTR reads (Table S1)) Second, for expanded VNTR lengths, also read length plays a more prominent role on coverage, since TR calling tools require reads to completely span the TR. Nevertheless for Subject10, which has an expanded allele and the shortest read length of all sequencing runs (since we tested the efficiency of PromethION on relatively degraded DNA), we could still span this largest allele with two sequencing reads. Moreover, despite sometimes low number of reads, their length estimates closely resemble the Southern Blotting length estimate. Hence, with ever-increasing flow cell performance, and more optimized DNA extractions and library preparations, TRs such as this ABCA7 VNTR can be called with high accuracy. Somatic variation has indeed not been observed for the ABCA7 VNTR, since Southern blotting always shows two clear bands (or 1 in case of homozygous length). Unstable TRs are more difficult to characterize with any technique and in the case of long-read sequencing, they would probably require more sequencing depth if accurate length estimations of all possible TR alleles are needed.

6. Seems to be very little information on the actual operation of "NanoSatellite" beyond that it is a DTW based algorithm. Similarly, what distances for the squiggle TR units were input into the unsupervised hierarchical clustering algorithm? I see Figure S6 says Ward distance from DTW distances, but it's still not clear to me what "DTW distance" means? I can assume the authors mean the DTW based length of the VNTR?

Author reply: To explain the NanoSatellite algorithm in more detail we now also added Figure S6 (with references on lines 170 and 477, and in the legend of Figure 1). DTW distance is directly generated by the “dtw” function in the “dtw” R-package for each alignment of two time series, with lower distances corresponding to higher similarities of these two time series. We basically use this distance measure in two scenarios: (1) finding the best match between a reference squiggle and raw Nanopore sequencing data to delineate tandem repeats and segment them in individual tandem repeat units, and (2) subsequently aligning these segmented TR unit squiggles against each other to identify nucleotide differences in the units (by using Ward’s method on DTW distance for unsupervised hierarchical clustering, as implemented in the “dtwclust” Rpackage). We aimed to provide more clarity about DTW distance on lines 506 – 508 and 523 – 527, and the legends of Figure S7 and S8.

7. Which 50 VNTRs were probed in NA19240? And what does a "preferential" call mean here?

Author reply: We now listed the 50 probed TRs and their classification in Table S3 and we made references to this table on lines 555 and 564 in the methods section. We made our (preferential) calls by scoring the TR length estimation plots. Aided by kmeans clustering using two centers, we evaluated how well these length estimations originating from both DNA strands cluster together and support diploid TR alleles. Figure 5 is an example of where we make a “NanoSatellite preferred” call, now partially validated by PCR, as explained below. NanoSatellite provides more evidence of a second, larger allele by showing tighter clustering of read lengths, and the presence of length estimations from negative DNA strands in the left panel. We now explain these preferential calls more clearly on line 560 – 563 in the methods and the legend of Figure 5.

8. And what is the "truth" set for NA19240? I understand that Southerns were used for ABCA7 - does such data exist for these other VNTRs in NA19240?

Author reply: As far as we know, there is no good publicly available truth set for TRs in NA19240. Southern blotting requires lengthy optimization procedures for each TR, which is too low throughput for this number of TRs. Hence to generate experimental validation we resorted to PCR amplification followed by gel electrophoresis for length estimation, and we added the resulting PCR determined TR lengths in Figure 5 and Figure S10. While we believe this method is the most suited for experimental validation, it has severe limitations. Due to the AT-rich or GC-rich nucleotide compositions and long possible TR lengths not all TRs and alleles can be successfully amplified and/or confidently called, which underscores the need for novel methods for TR genotyping, such as presented in this manuscript. For those TR alleles which we successfully PCR amplified, we see a good concordance with the length estimated by NanoSatellite and tandem-genotypes (Figure S10). We added PCR descriptions to the methods on line 565 – 573 and the results on line 226 – 227. For TRs without PCR validation (after extensive, but unsuccessful PCR optimization experiments), we used the clustering of repeat lengths obtained from independent sequencing reads as a measure of reliability. Particularly when lengths from both DNA strands cluster together, this provides strong evidence of accurate length estimation, as we observed from the Southern blotting-validated ABCA7 VNTR and PCR validated TRs. Furthermore also previous publications (Mitsuhashi et al., 2019, https://genomebiology.biomedcentral.com/articles/10.1186/s13059-019-1667-6) used a clustering of read lengths from both strands for accuracy and it correlates well with experimentally validated TR lengths when available. We elaborate on this in the Discussion on line 327 – 345.

9. A bit more about a "truth" set - ideally this algorithm would be verified against known truth. A plasmid with known amounts of synthetic repeats to verify the accuracy of the algorithm at the single molecule level is an important step that should be taken before other groups begin the implementation of NanoSatellite.

Author reply: Plasmids have been used in previous studies (e.g. tandem-genotypes was partly validated with plasmids, and we in turn compare NanoSatellite to tandemgenotypes), however, plasmids have limitations and do not guarantee good results in “real-life” WGS data, which is our main focus. They usually contain identical tandem repeat motifs, have different or no nucleotide modifications, lack the often repetitive complexity in the regions flanking the TR of interest, the maximum synthetic TR size is limited and often smaller than clinically relevant lengths, etc. One example of this is Ebbert et al., 2018, https://molecularneurodegeneration.biomedcentral.com/articles/10.1186/s13024-018- 0274-4: Long-read sequencing successfully characterized tandem repeats in C9orf72 ‘GGGGCC’ repeat plasmids. However, the largest plasmid they could construct contained 774 repeats (4.6kb), which is, as the authors note: “dramatically smaller than the expansion found in many affected carriers”. Furthermore, when they tested their approach on WGS from Sequel and MinION, they were confronted with low coverage in the TR region of interest. With the ABCA7 VNTR, we show that it is feasible to study ~10kb TR expansions using WGS long-read sequencing data. We elaborate on plasmids on line 331 – 335.

10. Also - I note that the authors do not discuss the TRs where "preferential" calls are made by tandem-genotypes or similar with both tools. This data suggests that NanoSatellite at these other VNTRs does not provide a significant advantage (based on numbers alone at least. Examination of Fig S9 bears this out to some extent - not knowing the "truth" of these VNTRs makes this data difficult to interpret.

Author reply: As described in the answer to question 8, we mostly rely on the extent to which read lengths from both DNA strands cluster in two alleles, since we and others have seen that this tends to correspond well with data from Southern blotting or PCR. Upon the requests of both reviewers, we now also added PCR lengths to Figure 5 and Figure S10. We also added a description of the PCR validation in the methods (line 565 – 573) and added a sentence to the results on line 226 – 227. We made a “preferential” call when either of the tools showed better read length clustering. The “similar” group indeed signifies that for some TRs, both tools produce the same results, which we consider advantageous. We discuss this point more clearly now on line 335. We fully agree with the reviewer that NanoSatellite does not always provide a significant advantage; see also our replies to comments 11 and 12 below.

We have made several adaptations in the text to clarify this point of view (line 351 – 358, line 359 – 363, line 374 - 377).

11. It would be beneficial to note some of the limitations of NanoSatellite. For example, the minimal length repetitive unit that can be accurately identified by NanoSatellite.

Author reply: The main limitation of NanoSatellite is that it requires a consistent pattern to reoccur. Except for homopolymers (TR unit length = 1), this requirement can be met by all TR unit lengths. However, as smaller TR units consist of less datapoints, DTW matching is more affected by “impurities” in the TR, such as nucleotide sequence differences, nucleotide modifications, and technical noise. Hence, TRs with short tandem motifs (length ≥ 2bp) can be studied with NanoSatellite, but they need to have a more consistent nucleotide composition than TRs with larger units. It is very difficult to define strict boundaries on these parameters, since they also depend on the sequence composition of the TR. Some nucleotide combinations / substitutions result in bigger electric current disruptions and therefore more recognizable squiggle patterns. We now elaborated more on the limitations of NanoSatellite in the discussion on line 351 – 360.

12. Lastly, the authors need to determine what impacts the performance of NanoSatellite and why it performs better on certain TRs. Groups utilizing this software tool need to be able to assess if it will perform well for a specific TR. In order for that to be possible, there need to be specific guidelines for the structure of the repetitive unit that is most compatible with this algorithm.

Author reply: In analogy to the previous question, the recognizability of the TR unit pattern determines the performance of NanoSatellite and this is determined by a combination of TR unit size, TR pureness, and TR nucleotide composition, which is unfortunately difficult to predict a priori. We would recommend users to first assess TRs of interest using conventional methods which benefit from faster performance for genome-wide analysis, when these algorithms do not provide accurate results, or when a second opinion is preferred, then NanoSatellite could be used to provide clarity. Further, the comparisons presented in Figure S11 (formerly S10) demonstrates that NanoSatellite may be particularly advantageous for repeats with higher GC content and larger number of repeat units. This is now discussed on line 360 – 363.

13. The authors say that there was "no clear relation between N50 read length and yield" - but this is clearly untrue as they state earlier that "lowest yield for the sequencing run with the longest DNA fragments". The longer N50 sample, in that case, has a markedly lower yield, but at the same time not the high N50 one would expect. I'm well aware that this is an ongoing problem with getting HMW DNA from extraction actually to the pore for sequencing, I just think this section needs more clarity.

Author reply: We agree with the reviewer. Originally, we meant to state that for those DNA samples for which we applied DNA shearing, we did not observe a correlation between the shearing length (which correlates to the read length and therefore also the N50) and yield, but this message was not clearly conveyed in the Discussion. Hence, we adapted the manuscript on line 254 – 255 to provide clarity.

**2^nd^ round**

**Reviewer 1**

Authors have addressed the comments well. I highly commend the authors for performing additional analysis and experiments to improve the manuscript. I have minor comments/clarifications to improve the clarity of the manuscript.

Line 115 - Subject01 is not included in FigureS3. I assume it is because of mixture of PromethION and MinION sequencing, but worth including a comment in figure legend.

Line 182 - Not clear why referenced to FigS5d instead of FigS5.

Line 187 - Expanded read detection is low for Guppy, however Guppy and NanoSatellite have similar accuracy, except for Subject05. Could the authors add more details on how the expanded read detection % in Table2 was determined.

Line 227 - Are the data for tandem-genotypes in figure 5, S9, S10 and S11 from Albacore+Tandem-genotypes or Guppy+Tandem-genotypes. It needs to be specified and ideally Guppy+Tandem-genotypes should be used, as it has high accuracy. The results should be updated accordingly.

Line 451 - Authors report using guppy v1.4.0, however the data they used is from guppy v2.3.5. Hence for clarity it would be better to mention the version used for analysis.

**Reviewer 2**

I'm satisfied with the manuscript in its current form after response to my comments (and those of the other reviewer).
